# Thermo/pH dual-responsive micelles based on the host–guest interaction between benzimidazole-terminated graft copolymer and β-cyclodextrin-functionalized star block copolymer for smart drug delivery

**DOI:** 10.1186/s12951-022-01290-3

**Published:** 2022-02-22

**Authors:** Floria Adeli, Farhang Abbasi, Mirzaagha Babazadeh, Soodabeh Davaran

**Affiliations:** 1 Department of Chemistry, Tabriz Branch, Islamic Azad University, Tabriz, Iran; 2 Institute of Polymeric Materials (IPM), Sahand University of Technology, Tabriz, Iran; 3 Research Center for Pharmaceutical Nanotechnology, Faculty of Pharmacy, Tabriz University of Medical Sciences, Tabriz, Iran

**Keywords:** Noncovalent polymeric system, Amphiphilic micelles, Drug delivery, Host–guest interaction, Smart nanocarriers, Star polymers

## Abstract

Novel temperature and pH dual-sensitive amphiphilic micelles were fabricated exploiting the host–guest interaction between benzimidazole-terminated PHEMA-*g*-(PCL-BM) and β-CD-*star*-PMAA-*b*-PNIPAM. The fabricated graft copolymer had a brush-like structure with star side chains. The micelles were utilized as dual-responsive nanocarriers and showed the LCST between 40 and 41 °C. The acidic pH promoted the dissociation of the PHEMA-*g*-(PCL-BM: β-CD-*star*-PMAA-*b*-PNIPAM) micelles. DOX.HCl was loaded into the core of the micelles during self-assembly in an aqueous solution with a high encapsulation efficacy (97.3%). The average size of the amphiphilic micelles was about 80 nm, suitable size for the enhanced permeability and retention effect in tumor vasculature. In an aqueous environment, these micelles exhibited very good self-assembly ability, low CMC value, rapid pH- and thermo-responsiveness, optimal drug loading capacity, and effective release of the drug. The biocompatibility was confirmed by the viability assessment of human breast cancer cell line (MCF-7) through methyl tetrazolium assay. DOX-loaded micelles displayed excellent anti-cancer activity performance in comparison with free DOX.

**Graphical Abstract:**

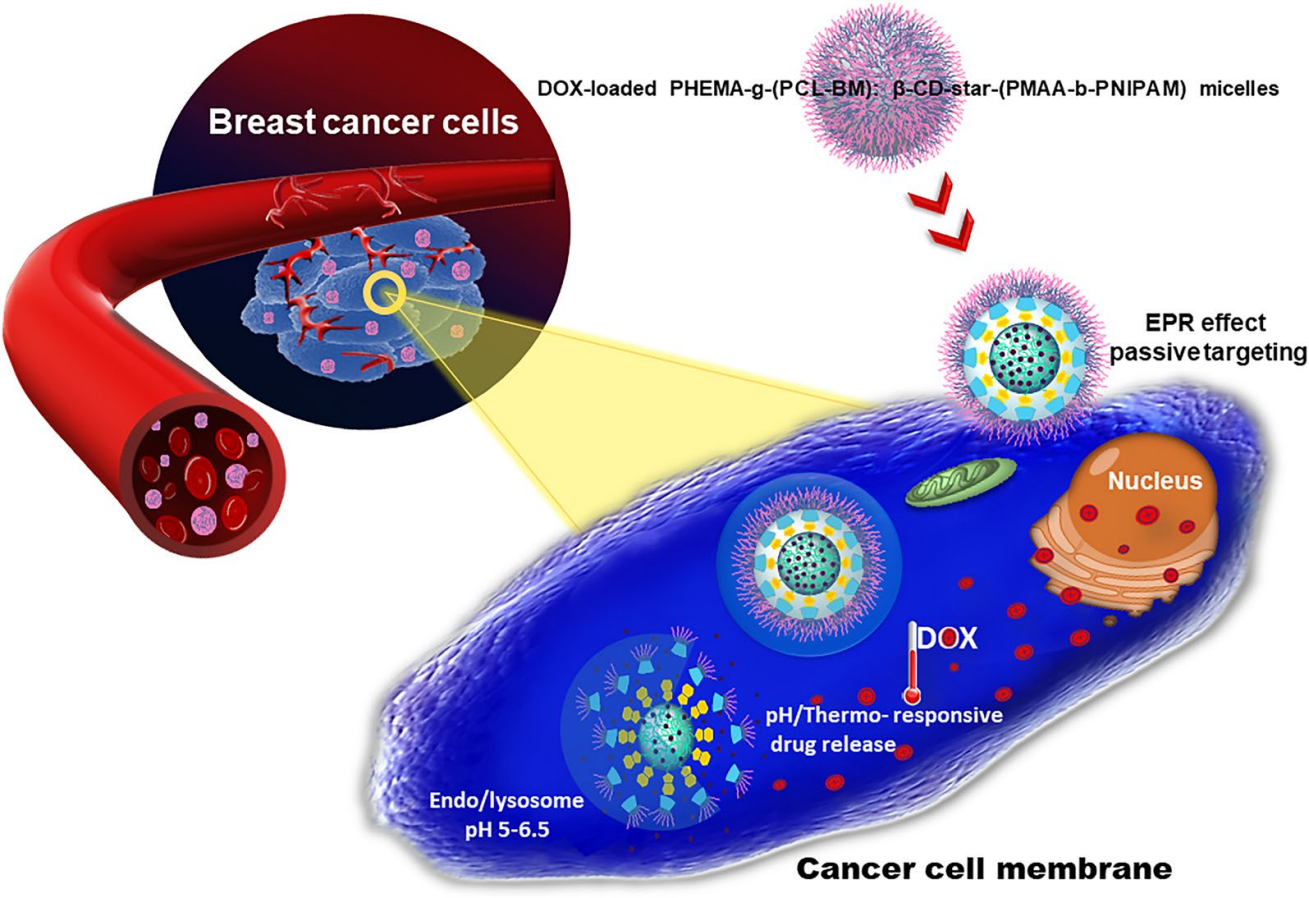

**Supplementary Information:**

The online version contains supplementary material available at 10.1186/s12951-022-01290-3.

## Highlights

- Thermo/pH dual-responsive micelles were formed in an aqueous solution based on host–guest interaction.
- The release of DOX was facilitated in acidic conditions and at temperatures above the LCST of the micelles.
- The drug-loaded micelles displayed higher antitumor activity than free DOX.

**Supplementary Information:**

The online version contains supplementary material available at 10.1186/s12951-022-01290-3.

## Introduction

In the field of drug delivery systems (DDS), nanomaterials are used to transport therapeutic drugs and diagnostic agents to target sites in a controlled manner [1]. The introduction of nanomaterials in drug formulation strategies is the main source of innovation in drug delivery [2]. The main reason for evaluating the drug delivery system is to increase pharmacological activity and reduce side effects. In targeted DDS, sometimes called smart drug delivery, the drug is transported to desired tissues, organs, cells, and subcellular organs; thus, its effect on vital tissues and adverse side effects can be minimized. In addition, DDS protects the drugs from degradation and clearance, improves the pharmacological activities of therapeutic drugs, and enhances drug concentration in target tissues. Therefore, lower doses of the drug are required. Dendrimers, liposomes, silicon or carbon materials, polymers, magnetic nanoparticles, and solid lipids nanoparticles are examples of nanocarriers that have been tested as DDS [3]. A large group of nanoparticles employed in DDS relies on amphiphilic copolymers to encapsulate therapeutic agents or drugs through self-assembly processes [4]. Amphiphilic copolymers, including block copolymers, brush polymers, hyperbranched polymers, dendrimers, etc., containing covalently attached hydrophobic and hydrophilic polymer chains are inclined to self-assembly in the presence of selective solvents [5, 6]. Graft copolymers, the general class of segmented copolymers, are comprised of numerous polymer side-chains grafted to a macromolecular backbone [7]. Noncovalent graft copolymers are one of the best and newest ways to produce smart supramolecular materials due to their high sensitivity in response to stimuli, relatively simple preparation, and rich topological structures. Various stimuli, including ultrasound, light, or microenvironmental changes in temperature and pH, could affect smart noncovalent graft copolymer [8, 9]. Overall, there are four ways to fabricate graft copolymers, including *grafting to* (the addition of previously prepared side chains to a backbone), *grafting from* (the polymerization of side chains from a macroinitiator backbone), *transfer to* (a combination of the *grafting to* and *grafting from* strategies), and *grafting through* (the polymerization of macromonomers) [7, 10]. Within the framework of each strategy, various polymerization techniques like ring-opening metathesis polymerization (ROMP), anionic and cationic polymerization, reversible-deactivation radical polymerization (RDRP), various coupling reactions (“click chemistry”), and miniemulsion polymerization are employed [11, 12]. The most popular reversible-deactivation radical polymerization techniques are reversible addition-fragmentation chain transfer polymerization (RAFT) and atom transfer radical polymerization (ATRP). These strategies not only enable the synthesis of graft copolymer with different compositions and functionality of side chains but also make good control over the molecular weight of the synthesized polymers [13, 14]. The choice and combination of these strategies and polymerization techniques make it possible to vary parameters of the resulting graft copolymers, including the size, grafting density, morphology, average degrees of polymerization of the backbone and side chains, and chemical composition. Most of the graft copolymers synthesized till now have linear side chains. However, there are few reports for star-like side chains [15–17]. Polymeric side-chains can be grafted to the main-chain (backbone) through covalent bonding or various noncovalent forces, including hydrogen-bonding interactions, ionic reactions, host–guest interactions, coordination bonding, and π–π stacking interaction [18, 19]. Among different types of noncovalent interactions, the host–guest interaction is a significant phenomenon to design amphiphilic polymers [12]. Host–guest chemistry describes complexes that are composed of two or more chemical moieties. They are linked together by noncovalent interactions and often show stimuli-responsiveness due to their dynamic properties [20]. Among various host molecules utilized for building up the host–guest inclusion complexes, cyclodextrins (CDs) have attracted extensive attention in the field of drug delivery due to their unique structure [21]. Cyclodextrins are cyclic oligosaccharides composed of multiple glucose subunits connected by α-1,4 glycosidic bonds. They have three native forms made up of six, seven, and eight glucose subunits in the ring (α-, β-, and γ-CDs, respectively) [22, 23]. Structurally, cyclodextrins as a host moiety are the truncated cone-like structure with internal hydrophobic cavities and external hydrophilic surface that usually accommodates the hydrophobic guest molecules to form inclusion complexes through noncovalent interactions [22]. The formation and dissociation of the inclusion complexes based on CD with a vast range of guest molecules are closely related to the environmental conditions (temperature and pH), the sizes of the guest and host molecules, as well as, the kinetic and thermodynamic properties of the complexes, which provide the possibilities for the stimuli-responsiveness of the host–guest systems [20].

To deliver therapeutic drugs into the systemic circulation, graft copolymers generally need to be amphiphilic and form micelles for drug encapsulation [24]. Polymeric micelles self-assembled are formed in an aqueous solution from amphiphilic copolymers. The shell of the micelle is formed by the hydrophilic moieties of amphiphilic molecules, whereas the micelle's core consists of hydrophobic segments of amphiphilic molecules [25]. These systems have a high potential for drug delivery applications. Amphiphilic micelles have widely been used to carry hydrophobic drugs, which are physically entrapped in and/or covalently linked to the hydrophobic core. The selection of desired polymers to design the micellar structures required in successful drug delivery is essential because the biological and physical characteristics of polymeric micelles depend upon the properties of starting blocks utilized for micelles preparation. These polymers influence the various micelles properties, for example, toxicity, bio-distribution, pharmacokinetics, and clinical compatibility [26].

**Scheme 1:**
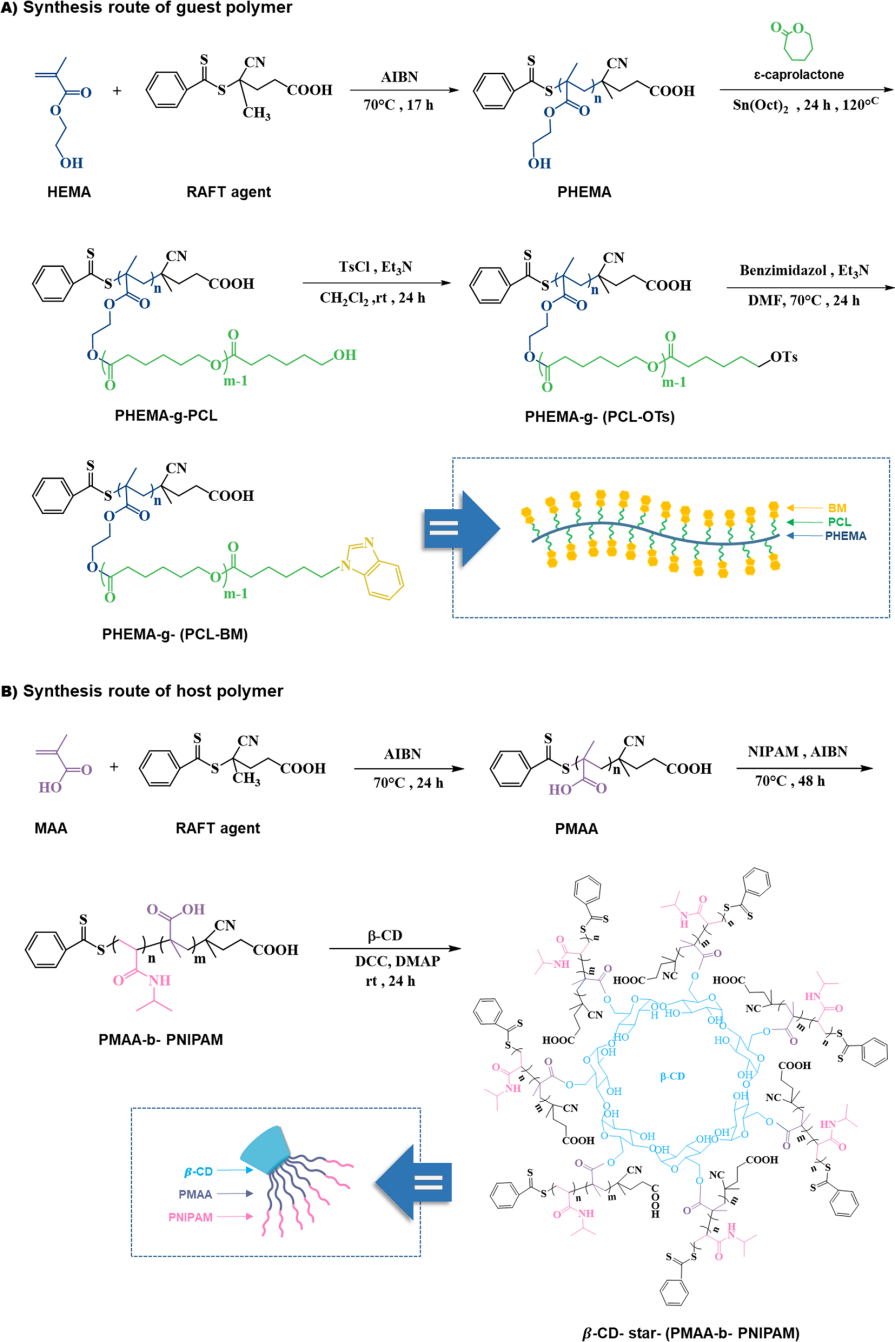
Schematic illustration and synthesis routes of PHEMA-*g*-(PCL-BM) graft copolymer and β-CD-*star*-(PMAA-*b*-PNIPAM) by RAFT, ROP, and coupling reactions

In this work, we fabricated a novel thermo/pH dual-responsive noncovalent graft copolymer with star-like side chains. The fabricated graft copolymer had a brush-like structure. As illustrated in Scheme 1, the polymer backbone with benzimidazole pendant groups was assembled with the star-like side chains via host–guest interaction between β-CD and benzimidazole segments. Subsequently, the supramolecular micelles were formed by self-assembly of the fabricated graft copolymers in an aqueous solution, and their architecture and properties were evaluated. The anticancer drug doxorubicin (DOX) as a model drug was employed to investigate the drug loading and in vitro drug release behavior from the prepared poly(2-hydroxyethyl methacrylate)-*graft*-[polycaprolactone-benzimidazole: β-cyclodextrin-*star*-poly(methacrylic acid)-*block*-poly(*N*-isopropylacrylamide)] [PHEMA-*g*-(PCL-BM: β-CD-*star*-PMAA-*b*-PNIPAM)] micelles. The potential of the resulting micelles as drug carriers was further investigated by cytotoxicity studies against MCF-7 human breast carcinoma cells.

## Experimental section

### Materials

4-Cyano-4-(phenylcarbonothioylthio)pentanoic acid as reversible addition-fragmentation chain transfer (RAFT) agent was prepared according to the reported procedure by Thang et al. [27]. 2-Hydroxyethyl methacrylate (HEMA, ≥ 97%, Merck) was dried over calcium hydride (CaH_2_) for 24 h, treated by passing through a column of neutral alumina for removal of inhibitor, and then was distilled under reduced pressure at 95 °C. ε-Caprolactone (ε-CL, 98%, Aldrich) was distilled under reduced pressure at 90 °C after being treated with CaH_2_ for 24 h. Tin(II) 2-ethylhexanoate (Sn(Oct)_2_, 95%, Aldrich) was used as received. β-Cyclodextrin (β-CD, 97%, Aldrich) was dried for 24 h in a vacuum oven before use. N-isopropylacrylamide (NIPAM, 99%, Acros) was recrystallized twice from *n*-hexane before use. N, N-dimethylformamide (DMF, ≥ 99.5%, Merck), as a solvent, was dried over CaH_2_ and distilled under reduced pressure before use. The initiator 2,2ʹ-azobisisobutyronitrile (AIBN, > 98%, Aldrich) was recrystallized twice from ethanol at 50 °C before use. Methacrylic acid (MAA, 99%, Merck) was distilled under vacuum before use. N,N′-dicyclohexylcarbodiimide (DCC, 99%, Aldrich), 4-(dimethylamino) pyridine (DMAP, 99%, Aldrich), *p*-toluenesulfonyl chloride (TsCl, ≥ 99%, Aldrich), benzimidazole (BM, ≥ 99%, Merck), diethyl ether (DEE, ≥ 99.5%, Merck), dimethyl sulfoxide (DMSO, ≥ 99.7%, Aldrich) and methanol (MeOH, ≥ 99%, Merck) were used without further purification. Dichloromethane (DCM, ≥ 99.8%, Merck) and triethylamine (TEA, 99%, Merck) were distilled over CaH_2_ just prior to use. Fetal bovine serum (FBS) and Trypsin–EDTA were purchased from Gibco (Life Technologies) (Carlsbad, USA). Phosphate buffer saline (PBS) and 3-(4,5-dimethylthiazol-2-yl)-2,5-diphenyltetrazolium bromide (MTT) were obtained from Sigma-Aldrich. MCF-7 human breast adenocarcinoma cells were obtained from the Pasteur Institute national cell bank of Iran (NCBI) (ATCC number: HTB-22, NCBI code: C135, Tehran, Iran). Doxorubicin hydrochloride (DOX.HCl, Sigma) was used without further purification.

### Preparation of noncovalent graft copolymer micelles

The noncovalent graft copolymer micelles were prepared via the following synthetic methods:

(i) Synthesis of poly(2-hydroxyethyl methacrylate) **(PHEMA)** as the backbone (1), synthesis of poly(2-hydroxyethyl methacrylate) grafted with polycaprolactone **PHEMA-g-PCL** (2), and synthesis of guest copolymer benzimidazole-containing PHEMA-*g*-PCL **(PHEMA-g-(PCL-BM))** (3);
(ii) Synthesis of poly(methacrylic acid) **(PMAA)** (4), synthesis of poly(methacrylic acid-*block*-N-isopropylacrylamide) (**PMAA-b-PNIPAM)** (5), synthesis of host polymer β-cyclodextrin-*star*-poly(methacrylic acid)-*block*-poly(N-isopropylacrylamide) (**β-CD-star-PMAA-b-PNIPAM)** (6);
(iii) Preparation of the micelles from β-CD-*star*-(PMAA-PNIPAM) and PHEMA-*g*-(PCL-BM).

#### Synthesis of PHEMA via RAFT polymerization

HEMA (4 mL, 32 mmol) and RAFT agent (30 mg, 0.1 mmol) were dissolved in dried Dimethylformamide (13 mL) in a dry round-bottom flask, and then the mixture was stirred under a N_2_ atmosphere at 0 °C for at least 1 h. The reaction was started by adding AIBN (1.7 mg, 0.01 mmol) and allowed to proceed at 70 °C for about 17 h. The experiment was stopped by cooling the glass flask in an ice bath. The final reaction solution was precipitated with cold diethyl ether and then, dried under vacuum at 25 ºC. $\overline{\mathrm{M} }$_n_ ~ 9260 g mol^−1^. ATR-FTIR (cm^−1^): 3400, 2944, 2883, 1720, 1658, 1451, 1388, 1273, 1152, 1074, and 1023.

#### Synthesis of PCL-grafted PHEMA with (PHEMA-*g*-PCL)

The PHEMA-*g*-PCL was synthesized by the ring-opening polymerization (ROP) as follows. PHEMA as the macroinitiator (0.1 g, 0.76 mmol − OH) was dissolved in 2 mL of dried DMF in a 50 mL flame-dried flask under a N_2_ atmosphere, and stannous octoate (8.91 mg, 0.022 mmol) as catalyst was added to the flask. The solution was stirred at 90 °C for 30 min. Next, ɛ-caprolactone (2.5 g, 22.09 mmol) was added to the reaction mixture, and the temperature was raised to 125 °C, and the reaction was continued for 12 h. Finally, the mixture was dissolved in dichloromethane, and precipitated in methanol three times. The precipitated polymer was collected by filtration and drying in vacuo. $\overline{\mathrm{M} }$_n_ ~ 166,500 g mol^−1^. FTIR (KBr, cm^−1^): 3419, 2866 − 2946, 1726, 1619, 1472,1420,1371,1297, 1244, 1189, 1106, and 1045.

#### Synthesis of benzimidazole-containing PHEMA-*g*-PCL [PHEMA-*g*-) PCL-BM)]

PHEMA-*g*-(PCL-BM) was synthesized in two steps. In the first step, a solution of 0.4 g (0.16 mmol − OH) PHEMA-*g*-PCL in 15 ml anhydrous DCM and 0.2 ml (1.6 mmol) TEA was poured in a round-bottom flask equipped with a gas inlet/outlet, and a magnetic stirrer. The solution was mixed with *p*-toluenesulfonyl chloride (0.3 g, 1.6 mmol)) under a nitrogen atmosphere at 0 °C. Then, the reaction was allowed to warm up to room temperature and proceed at ambient temperature for 24 h. After filtration, the clear solution was poured into diethyl ether. Then, the resulting tosylated copolymer was collected by centrifugation and dried at 35 °C under reduced pressure for 24 h. With the appropriate leaving group in the substrate (− OTs), benzimidazole can be coupled to PHEMA-*g*-PCL by a nucleophilic substitution reaction. In the next step, benzimidazole (0.131 g, 1.1 mmol) and TEA (0.15 ml,1.1 mmol) were dissolved in 2 ml of anhydrous DMF inside a dry round-bottom and nitrogen-filled flask. Subsequently, a solution of PHEMA-*g*-(PCL-OTs) (0.3 g, 0.11 mmol) in anhydrous DMF (2 mL) was injected by a syringe into the flask under a nitrogen atmosphere. The reaction mixture was stirred at 70 °C for 24 h. Finally, the mixture was precipitated twice from diethyl ether and then, dried under vacuum at room temperature. Scheme 1A shows the reactions for the preparation of PHEMA-*g*-(PCL-BM) as the guest polymer. $\overline{\mathrm{M} }$_n_ ~ 172,000 g mol^−1^. FTIR (KBr, cm^−1^): 3417−3480, 2886−2945, 1726, 1620, 1473, 1372, 1244, 1190, 1041 and 732.

#### Synthesis of PMAA via RAFT polymerization

Methacrylic acid (1 g, 11.6 mmol) and RAFT agent (0.15 g, 0.53 mmol) were dissolved in dried DMF (13 mL) inside a dry round-bottom flask. The solution was stirred under a N_2_ atmosphere at 0 °C for at least 1 h. The reaction was started by adding AIBN (2 mg, 0.012 mmol) into the flask and allowed to proceed at 75 °C. After 8 h, the reaction mixture was quenched in an ice bath, precipitated with an excess amount of diethyl ether, and then, dried under vacuum at 25 ºC. $\overline{\mathrm{M} }$_n_ ~ 2085 g mol^−1^. ATR-FTIR (cm^−1^): 2929, 1722, 1641, 1439, 1386, 1255, 1166, and 1104.

#### Synthesis of PMAA-*b*-PNIPAM

A typical polymerization procedure was as follows: Poly(methacrylic acid) as macro-RAFT agent (PMAA, 0.5 g, 0.24 mmol) and NIPAM (0.4 g, 3.5 mmol) were dissolved in dried DMF (8 ml) inside a dry flask, and then the mixture was stirred at 0 °C and purged with N_2_ atmosphere for at least 60 min to degas the mixture. After which time, the reaction was started by adding AIBN initiator (2 mg, 0.012 mmol) in the flask and was allowed to continue at 85 °C for about 48 h. At the end of this time, the final reaction solution was precipitated with an excess amount of cold diethyl ether three times and then, dried under vacuum at ambient temperature. $\overline{\mathrm{M} }$_n_ ~ 5480 g mol^−1^. FTIR (KBr, cm^−1^): 3439, 1700, 1640, 1439, 1386, 1255, 1166, and 1104.

#### Synthesis of β-CD-*star*-PMAA-*b*-PNIPAM

β-CD-*star*-(PMAA-*b*-PNIPAM) copolymers were prepared by coupling β-cyclodextrin to PMAA-*b*-PNIPAM. In a typical procedure, PMAA-*b*-PNIPAM (0.3 g, 0.054 mmol) was dissolved in 5 ml of anhydrous DMF, and the mixture was purged with N_2_ for 10 min at 25 °C. Then, anhydrous β-CD (0.15 g, 0.13 mmol) was added and stirred for complete dissolution. Subsequently, DCC (0.1 g, 0.48 mmol) and DMAP (0.01 g, 0.081 mmol) were added and stirred under a nitrogen atmosphere at ambient temperature for 24 h. Then, the byproduct dicyclohexylcarbodiurea (DCU) was removed by filtration, and the product was obtained after precipitation into a large amount of cold diethyl ether and dried under vacuum conditions at room temperature. Scheme 1B shows the preparation representation of β-CD-*star*-(PMAA-*b*-PNIPAM) as the host polymer. $\overline{\mathrm{M} }$_n_ ~ 39,500 g mol^−1^. FTIR (KBr, cm^−1^): 3442, 2228–2978, 1759, 1717, 1631, 1565, 1027, and 948.

#### Preparation of noncovalent graft copolymer micelles via host–guest interaction

The noncovalent graft copolymer micelles were fabricated through host–guest interaction between β-CD-*star*-(PMAA-*b*-PNIPAM) host polymer and PHEMA-g-(PCL-BM) gest polymer. Briefly, β-CD-*star*-(PMAA-*b*-PNIPAM) (15 mg) was dissolved in 1 ml of dimethyl sulfoxide (DMSO) and added dropwise to the solution of PHEMA-*g*-(PCL-BM) (5 mg) in DMSO (1 ml) under vigorous stirring. Then, the mixture solution was kept under magnetic stirring at ambient temperature for 24 h to ensure complete inclusion. Subsequently, the polymer solution was added dropwise into 5 ml of deionized water, and stirred for another 2 h to ensure the formation of supramolecular micelles at ambient temperature. Then the solution was transferred into a dialysis membrane bag with a molecular cut-off (MWCO) of 10 kDa and dialyzed for 24 h against deionized water to remove DMSO.

### Preparation of DOX-loaded noncovalent graft copolymer micelles

**Scheme 2:**
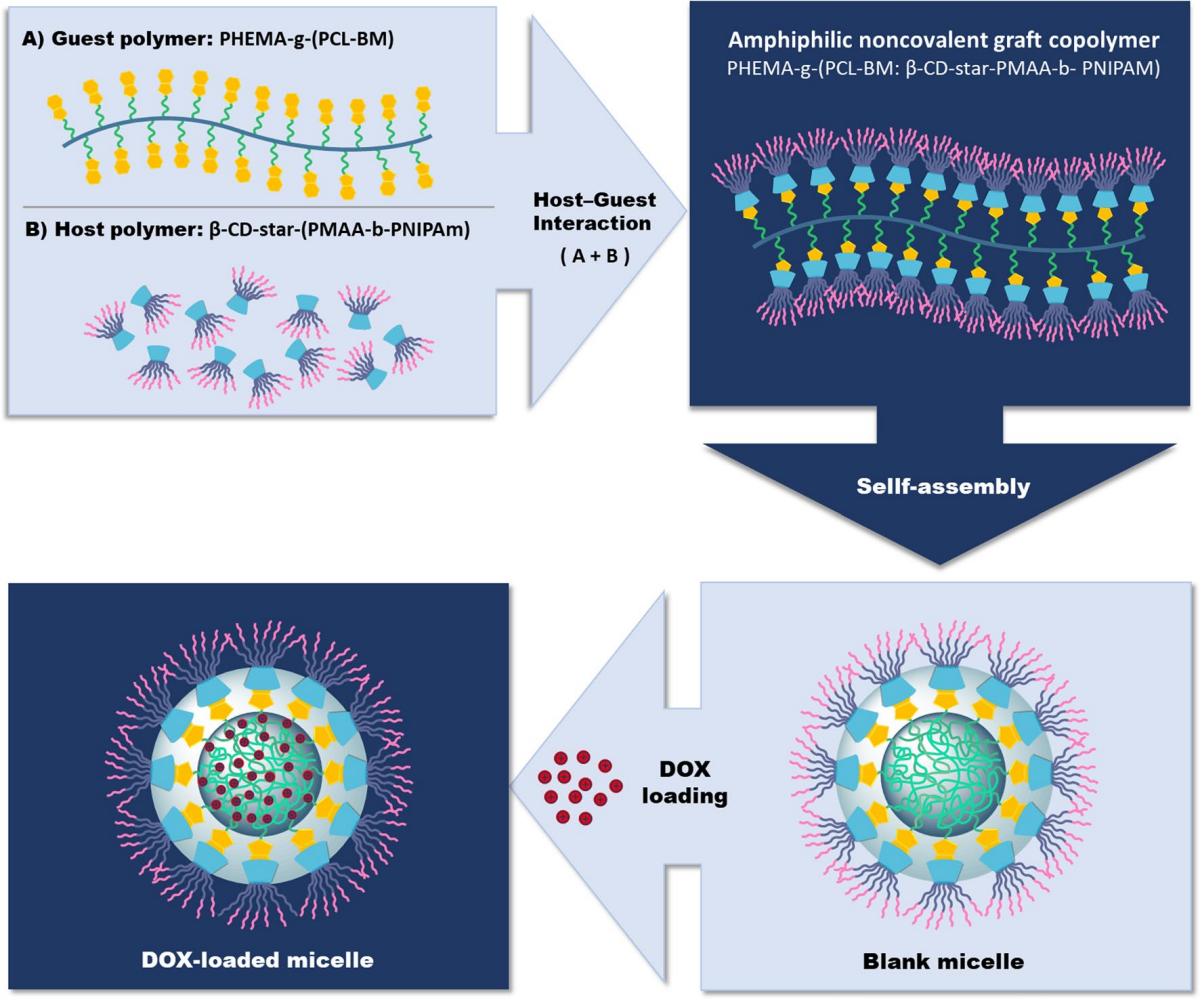
Schematic representation of the formation of temperature/pH dual-sensitive drug-loaded micelles from amphiphilic noncovalent graft copolymer PHEMA-*g*-(PCL-BM: β-CD-*star*-PMAA-*b*-NIPAM)

Drug-loaded noncovalent graft copolymer micelles were prepared using DOX.HCl as an anticancer model drug at room temperature, similar to the blank micelles. Typically, β-CD-*star*-(PMAA-*b*-PNIPAM) (60 mg) was dissolved in 1 ml of dimethyl sulfoxide (DMSO) and added slowly into the solution of PHEMA-*g*-(PCL-BM) (20 mg) in DMSO (1 ml) under vigorous stirring. The mixture was kept under magnetic stirring at 25 °C for 24 h to ensure complete inclusion. After this time, a certain amount of DOX.HCl (8 mg) was added slowly into the above inclusion solution and stirred at 25 °C in dark conditions for 48 h. Then, the mixture solution was added dropwise in 5 mL of deionized water. After being stirred for 2 h, the mixture solution was dialyzed against deionized water (MWCO 10 kDa) for another 24 h and then, centrifuged at 12,000 rpm for about 20 min to remove free DOX. At the end of this time, the supernatant was kept to measure the concentration of encapsulated drug using the calibration curve of the DOX, as shown in Additional file 1: Figure S1. The DOX-loaded micelles were lyophilized and stored at 4 °C (Scheme 2).

### Determination of drug loading content (LC) and encapsulation efficiency (EE)

In the first stage, the calibration curves of DOX in phosphate buffer saline (pH 7.4, 5.7, 4.5) were constructed by plotting the absorbance value versus the concentration of DOX (Additional file 1: Figure S1). After loading the drug in the polymeric micelles by dialyze method, the mixture solution was centrifuged at 12,000 rpm for about 20 min at 25 °C to remove the free DOX. At the end of this time, the supernatant passed through an Amicon Ultra centrifugal filter (100 KDa cutoff, Millipore; USA) to remove the excess micelle. In order to analyze the loading efficiency of DOX in the fabricated micelles, the concentration of unloaded drug was determined using a UV–Vis spectrophotometer at wavelength of 480 nm and replacing its absorption in the trend line equation from the calibration curve. The DOX-encapsulation efficiency (EE%) and loading content (LC%) were calculated according to the following equations:

$$\mathrm{EE }(\mathrm{\%})=\frac{\mathrm{Amount \, of \, loaded \, drug}}{\mathrm{Total \, drug}}\times 100$$

$$\mathrm{Amount \, of \, loaded \, drug}=\mathrm{Total \, drug }-\mathrm{unloaded \, drug}$$

$$\mathrm{LC }\left(\mathrm{\%}\right)=\frac{\mathrm{Mass \, of \, the \, loaded \, drug \, in \, the \, nanocarrier}}{\mathrm{ Nanocarrier \, mass}}\times 100$$

The DOX-encapsulation efficiency was calculated to be 97.3%.

### In vitro temperature- and pH-dependent release study

In vitro release behavior of drug from DOX-loaded micelles was performed using the sample and separate method [28, 29]. For this purpose, the drug-loaded micelles were dispersed in a vessel containing a certain amount of release media, and the release of the drug was assessed over certain times. The release study of the drug from the micelles was investigated in phosphate-buffered saline (PBS) at various pH (7.4, 5.7, and 4.5) and temperature values (37 and 42 °C). In this procedure, 4 mg freeze-dried DOX-loaded micelles were dispersed in buffer solution (2 ml) and placed into the incubator shaker that provided continuous rotation and the constant temperature. The drug release was assessed by sampling the release media (supernatant). At the definite time intervals, 1 ml of release media was taken and centrifuged at 12,000 rpm for 10 min. After sampling, an equal amount of fresh PBS is added to the set-up so that sink conditions are maintained for the duration of the in vitro release study. The DOX amount released from the micelles was detected by the UV–Vis spectrophotometer and absorbance values were recorded at the maximum wavelength of the drug (λ_max_ = 480 nm). The cumulative percentage of drug released over time was calculated using the following Eq. (28):

$$\mathrm{Cumulative \, release }=\frac{{\mathrm{c}}_{\mathrm{i}}{\mathrm{v}}_{\mathrm{t}}+\sum {\mathrm{c}}_{\mathrm{i}-1}{\mathrm{v}}_{\mathrm{i}}}{\mathrm{t}}\times 100$$

where v_t_ is the total volume of release solution, v_i_ is the sample volume, c_i_ is the concentration of drug in the released solution at the time i, and t is the concentration of the total drug (µg/ml). To avoid statistical error, the evaluation was performed in triplicate for each sample.

### Cell culture

The human breast cancer cell line (MCF-7) was purchased from NCBI (National Cell Bank of Iran, Pasteur Institute) and maintained in RPMI 1640 medium augmented with 100 IU/ml penicillin, 10% fetal bovine serum (FBS), and 100 μg/ml streptomycin at 37 °C in a humidified atmosphere containing 5% CO_2_. MCF-7 cells at a concentration of 0.5 × 10^6^ were grown in a 25 cm^2^ culture plate in 5 ml of complete culture medium. When the MCF-7 cells population reached 70% confluency, trypsin–EDTA was added to the culture plate and placed for 5 min in the incubator to detached cells. For neutralizing the trypsin–EDTA activity, 2 ml FBS containing media was utilized. The harvested cells were centrifuged at 900 rpm at room temperature for 5 min. Finally, the cells with fresh culture medium were seeded in 96-well flat-bottomed culture plates with a cell density of 15 × 10^3^ cells per well and incubated the plate at 37 °C in a 5% CO_2_ humidified incubator for 48 h.

### In vitro cytotoxicity assay

The cytotoxicity test is one of the most important markers for biological assessment in vitro studies [30]. The in vitro cytotoxicity of the blank polymeric micelles, free DOX, and DOX-loaded micelles against MCF-7 cells were investigated in the concentration ranges between 0.3 to 50 μg/ml using 3-(4,5-dimethyl-2-thiazolyl)-2,5-diphenyl-2-H-tetrazolium bromide (MTT) assay, as shown in Fig. 6. Briefly, cancer cells were seeded in a 96-well flat-bottomed culture plate at an initial density of 1 × 10^4^ cells per well and allowed to attach for 24 h. Then, MCF-7 cells were exposed to various concentrations of blank polymeric micelles, free DOX, and DOX-loaded micelles for 48 h. After incubation 48 h, the culture medium of the incubated plates was detached and 50 μl MTT solution followed by 150 μl fresh cultivation medium added to the wells and then incubated for 4 h. In continue, the culture medium was exchanged with 150 μl of dimethyl sulfoxide (DMSO) to dissolve the formazan crystals formed by viable cells. Finally, the optical density (OD) of the solution was measured by a microplate reader (Elx808, Biotek, USA) at a wavelength of 570 nm. The percentage of cell viability was obtained according to the following equation:

$$\mathrm{Cell \, viability }(\mathrm{\%}) =\frac{{\mathrm{OD}}_{\mathrm{t}}}{{\mathrm{OD}}_{\mathrm{b}}}\times 100\mathrm{\%}$$

In this equation, OD_t_ and OD_b_ are the optical density values of surviving cells treated with test group and blank control, respectively. The results represent the average values of three measurements [31].

### Morphological assessment of apoptotic cells by DAPI Staining

In order to visualize the apoptotic effect of DOX formulations against MCF-7 cells, the nuclei of the cells were stained with 4,6-diamidino-2-phenylindole (DAPI). In the present study, MCF-7 cells were seeded onto a 6-well plate at 2 × 10^5^ cells per well in 2 ml of medium and cultured for 24 h at 37 °C. After this time, the culture medium was replaced with fresh medium containing free DOX, and DOX-loaded micelles in which their concentration was around IC50, and 50 μg/ml. After incubating at 37 °C for 48 h, the cells were fixed with fresh 4% paraformaldehyde and then permeabilized with Triton X-100 (0.1% in PBS). Cells were finally stained using DAPI and allowed to stand for 15 min in a dark condition. Lastly, morphological changes were viewed using fluorescence microscopy at 200× and 400× magnification, and excitation at 405 nm for DAPI.

## Characterization

### Fourier-transform infrared (FTIR) spectrometry

FTIR spectra were recorded on a Tensor 27 spectrophotometer (Bruker, Germany) in the wave number range 4000−400 cm^−1^. The specimens were prepared using the KBr pellet method.

### Attenuated total reflection Fourier transform infrared (ATR-FTIR) spectroscopy

ATR-FTIR spectra were recorded in the 4000 to 400 cm^−1^ wavenumber region using Bruker Tensor 27 FTIR spectrometer equipped with the ATR platinum module. The ATR part is composed of a diamond disc as an internal reflection element. The sample was placed on the ATR crystal, and the ATR-FT-IR spectrum was scanned at a spectral resolution of 4 cm^−1^.

### Nuclear magnetic resonance (NMR) spectroscopy

^1^H NMR spectra of samples were recorded on a Proton nuclear magnetic resonance (300 mHz) spectrometer (Bruker, AVANCE III) with DMSO-d6 and CDCl_3_ as solvents. The chemical shifts were relative to tetramethylsilane.

### Two-dimensional nuclear overhauser effect spectroscopy (2D-NOESY) NMR spectra

2D-NOESY NMR experiments were performed at 400 MHz in DMSO-d6 as solvent on a Bruker Avance III NMR spectrometer.

### Fluorescence spectroscopy

Fluorescence spectroscopy is a powerful tool and is becoming a routine method for studying the formation of CD inclusion complexes with fluorescent guests in solution. Because fluorescence spectroscopy is a high sensitivity, so allows working with very low guest concentrations [32]. The fluorescent probe technique was employed to determine the critical micelle concentration (CMC) of PHEMA-*g*-(PCL-BM: β-CD-*star*-PMAA-*b*-PNIPAM) micelles using pyrene as a fluorescence probe (RF-5301; Shimadzu Co, Kyoto, Japan). In brief, a predetermined amount of pyrene solution in acetone was added into a series of volumetric flasks, and acetone was evaporated under nitrogen stream at room temperature for 5 h. A series of the copolymer solutions at different concentrations ranging from 1.0 × 10^–4^ − 1.0 mg ml^−1^ were added to the flasks, while the concentration of pyrene in each flask was fixed at a constant value 6 × 10^−7^ M (the solubility of pyrene in water). After keeping all the samples at room temperature overnight, they were subjected to the assay of fluorescence intensity at an excitation wavelength of 343 nm. In order to calculate the CMC value, the curve of fluorescence intensity ratio (I_384_/I_373_) was plotted versus logarithmic concentration (log C) (C in mg ml^−1^).value was determined from the intersection of the two tangent lines created in the graph.

### Dynamic light scattering (DLS) measurements

Particle size and zeta-potential of micelles in the aqueous solution was measured using dynamic light scattering (DLS) (Zetasizer Nano ZS90; Malvern Instruments, UK). The samples were readied with a concentration of 0.1wt%.

### Transmission electron microscopy (TEM)

The TEM image of the prepared micelles was taken using Zeiss, Leo 906 microscope 100 kV working voltage. A drop of micelle solution (0.2 mg ml^−1^) was deposited onto a 230-mesh copper grid coated with carbon and allowing the sample to dry at room temperature before making TEM measurements.

### Field emission scanning electron microscope (FESEM) analysis

Morphological analysis was also conducted through field emission scanning electron microscopy (FESEM) (Hitachi, S4160) to validate the data obtained by TEM. After the samples had been prepared, they were settled on a stub containing carbon and subsequently covered with a thin layer of gold.

### Determination of lower critical solution temperature (LCST)

The temperature-responsive behavior was investigated by the turbidimetry method. Turbidity variation of the samples at various temperatures was measured using UV–visible spectrophotometer at a wavelength of 600 nm. For this purpose, the samples were heated from 30 to 50 °C. The LCST was determined from the transmittance of samples containing polymeric micelles as a function of temperature.

### Atomic force microscopy (AFM)

The AFM analysis was performed using a JPK-NanoWizard II scanning probe microscope operated in tapping mode. The thin films were obtained by spin-coating the polymer solutions (0.1 mg ml^−1^) on transparent mica.

### Statistical analysis

Statistical analyses of the in vitro cellular works were conducted using a Graph-Pad Prism version 8. All experiments were performed in the triplicated, and the data were expressed as mean ± standard deviation (SD). Data ^1^HNMR were analyzed using the MestReNOVA analysis. Data were analyzed for statistical significance using the one-way ANOVA analysis. A statistically significant difference was reported if the p-value was less than or equal to 0.05.

## Results and discussion

Nanotechnology paves ways for the design and development of advanced drug delivery systems having control and ultra-precision over the release of drugs, which dramatically change the novel strategies of drug delivery. Various approaches, such as encapsulation, targeting molecules or specific biomarkers, and using artificial nanocarriers are adopted to ensure controlled and targeted (or smart) drug delivery. Biodegradable and intelligent polymeric materials pave the way for controlled, targeted drug delivery in modern therapeutics owing to their unique physical and chemical properties, excellent bioavailability, controlled release, biocompatibility, and less toxicity [33, 34]. This research aimed to design and development of thermo/pH dual responsive noncovalent graft copolymer micelles based on the host–guest interaction for enhanced drug delivery applications.

### Synthesis and characterization of guest polymers

The guest polymers were prepared in three sequential steps, as explained in Scheme 1. In the first step, PHEMA as the backbone, was synthesized by the RAFT polymerization, using 4-cyano-4-(phenylcarbonothioylthio)pentanoic acid as a chain transfer agent. In the second step, PHEMA-*g*-PCL was synthesized by ring-opening polymerization of CL in the presence of PHEMA as a macroinitiator and Sn(Oct)_2_ as a catalyst. In the last step, PHEMA-*g*-PCL-OTs was obtained by tosylation of PHEMA-*g*-PCL in the presence of TEA, and then, guest copolymer benzimidazole-containing (PHEMA-*g*-(PCL-BM)) was synthesized via replacement of the tosyl moiety with BM in the presence of TEA. The chemical structures of the guest polymers were characterized by FTIR analysis (Additional file 1: Figure S2).

**Fig. 1:**
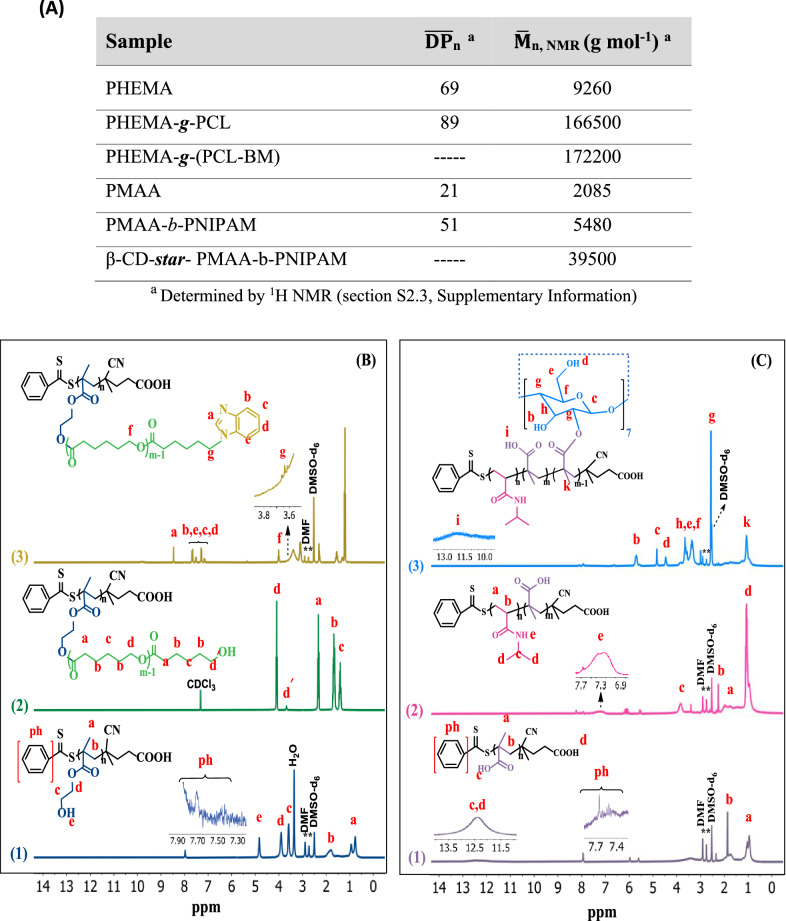
**A** Characteristics of PHEMA-*g*-(PCL-BM) and β-CD-*star*- PMAA-*b*-PNIPAM. **B** ^1^H NMR spectra of PHEMA in DMSO-d6 (1), PHEMA-*g*-PCL in CDCL_3_ (2), and PHEMA-*g*-(PCL-BM) in DMSO-d6 (ppm) (3). **C** ^1^H NMR spectra of PMAA in DMSO-d6 (1), PMAA-*b*-PNIPAM in DMSO-d6 (2), and β-CD-*star*- (PMAA-*b*-PNIPAM) in DMSO-d6 (ppm) (3)

The synthesized PHEMA, PHEMA-*g*-PCL, and PHEMA-*g*-PCL-BM copolymer were further characterized using ^1^H NMR spectroscopy (Fig. 1B). In the ^1^H NMR spectrum of the PHEMA (Fig. 1B1), the proton peak at 7.4 –7.93 belonged to aromatic protons of the RAFT agent. The methylene and methyl protons of the PHEMA skeleton were appeared at 1.77–1.92 and 0.77–0.93 ppm, respectively. The protons of the –OCH, and –CHOH groups were observed at 3.88 and 3.58 ppm, respectively. Moreover, the chemical shift at 4.81 ppm was attributed to the hydroxyl groups of the PHEMA. The average degree of polymerization ($\overline{\mathrm{DP} }$_n_) of the synthesized PHEMA was determined to be ~ 69 by ^1^H NMR data (Additional file 1: Figure S2.3a).

A typical ^1^H NMR spectrum of the copolymer PHEMA-*g*-PCL with the assignment is shown in Fig. 1B2. The major resonance peaks (a–d) were attributed to PCL segments in PHEMA-*g*-PCL. The methylene proton signal (d′, δ = 3.6 ppm) indicated that PCL was terminated by hydroxyl groups. The feed molar ratio of [CL] to [OH] of PHEMA was 25:1. The average degree of polymerization for the PCL segments in PHEMA-g-PCL copolymers was calculated by ^1^H NMR from the integration of the signals of methylene protons (peak d, δ = 4.1 ppm) to the terminal methylene protons (peak d′, δ = 3.6 ppm) (see Fig. 1A). The molecular weight of the PHEMA-g-PCL ($\overline{\mathrm{M} }$_n_ ~ 166,500 g mol^−1^) was determined from the ^1^H NMR data (Additional file 1: Figure S2.3(b)). The chemical structure of PHEMA-g-(PCL-BM) copolymer was characterized by ^1^H NMR, as displayed in Fig. 1B3. The major resonance peaks at 7.1–7.7 ppm corresponded to benzene protons, and the chemical shift at 8.4 ppm attributed to imidazole proton were completely appeared, which showed the benzimidazole molecules were conjugated onto PHEMA-*g*-PCL [35]. The results proved the successful synthesis of PHEMA-*g*-(PCL-BM) copolymer. The coupling efficiency of PHEMA-*g*-(PCL-BM) was calculated to be 70% based on the relative integral values of the methylene signal on the PCL (–CH_2_OCO–, 3.9–4.1 ppm) and that connected with benzimidazole (–CH_2_CH_2_–N_benzimi_, 3.6 ppm) from the ^1^H NMR spectra (see Eq. (4), Additional file 1: Figure S2.3(c)) [36]. In addition, the number average molecular weight of the synthesized PHEMA-*g*-(PCL-BM) was calculated from the ^1^H NMR data (Additional file 1: Figure S2.3(c)).

### Synthesis and characterization of host polymers

The dual responsive host polymer β-CD-*star*-(PMAA-*b*-PNIPAM) was prepared through three sequential steps as shown in Scheme 1. In the first step, the PMAA homopolymer was synthesized through RAFT polymerization of methacrylic acid monomer using AIBN as initiator and 4-cyano-4-(phenylcarbonothioylthio)pentanoic acid as a chain transfer agent at 75 °C. In the second step, to synthesize of PMAA-*b*-PNIPAM copolymers, RAFT-synthesized PMAA was used as a macromolecular RAFT agent and AIBN as initiator at 85 °C. In the last step, for the synthesis of β-CD-*star*-PMAA-*b*-PNIPAM, the esterification reaction of β-CD with PMAA-*b*-PNIPAM was carried out in the presence of DCC and DMAP at room temperature. The success of chemical synthesis of the PMAA, P(MAA-*b*-NIPAM), and β-CD-*star*-(PMAA-*b*-PNIPAM) could be confirmed from the FTIR spectral results (Additional file 1: Figure S2.2), which were further supported by ^1^HNMR spectra.

The synthesized PMAA, PMAA-*b*-PNIPAM, and β-CD-*star*-PMAA-*b*-PNIPAM copolymer were further characterized through ^1^H NMR spectroscopy (Fig. 1C).

In the ^1^H NMR spectrum of the PMAA, the proton peaks at 0.81–1.14 ppm (a), 1.6–1.9 ppm (b) are assigned to the methyl and methylene protons of the PMAA, respectively (Fig. 1C(1)) [37]. In addition, the aromatic protons of the RAFT agent and hydroxyl group give peaks at 7.4 –7.93 ppm (ph) and 12.4 ppm (c), respectively [38]. The average degree of polymerization of the synthesized PMAA was determined to be ~ 21 by ^1^H NMR data (Additional file 1: Figure S2.3(d)).

The ^1^H NMR spectrum of PMAA-*b*-PNIPAM was shown in Fig. 1C2. The proton peaks at 0.73–1.17 ppm (d), 1.6–2.13 ppm (a), and 2.18–2.3 ppm (b) in the ^1^HNMR spectrum of PMAA-*b*-PNIPAM copolymer corresponded to the methyl, methylene, and methine groups existing on the carbon skeleton of the copolymer. Moreover, the –CH–NH protons of the PNIPAM block (c) were observed at about 3.86 ppm. $\overline{\mathrm{DP} }$_n_ of PNIPAM was calculated by comparing the composition of PNIPAM block with PMAA block using the integral area of their respective protons, as indicated in Fig. 1C (see Eq. (8), Additional file 1: Figure S2.3(e)). $\overline{\mathrm{M} }$_n, NMR_ of PNIPAM was calculated, and molecular weight of the prepared PMAA-*b*-PNIPAM diblock copolymer was obtained by combining $\overline{\mathrm{M} }$_n, NMR_ of PMAA and PNIPAM blocks (see Fig. 1A). The molecular weight of the PMAA-*b*-PNIPAM was determined to be 5480 g mol^−1^ (Additional file 1: Figure S2.3(e)).

The ^1^H NMR spectrum of the β-CD-*star*- PMAA-b-PNIPAM was shown in Fig. 1C3. After the reaction, the new peaks at 2.5 ppm (g), 3.2−3.6 ppm (h, e, f), 4.4 ppm (d), 4.8 ppm (c), and 5.5−5.8 ppm (b) are assigned to the β-cyclodextrin group. According to the integral of C(k)H and C(c)H, the number of carbonyl groups is calculated as ~ 7 per β-CD molecule. The molecular weight of the β-cyclodextrin-*star*-PMAA-b-PNIPAM was calculated to be ~ 39,500 g mol^−1^ (Additional file 1: Figure S2.3(f)).

### Characterization of noncovalent graft copolymer micelles

Due to their amphiphilic nature, in the aqueous solution, PHEMA-*g*-(PCL-BM):(CD-*star*-PMAA-*b*-PNIPAM) molecules can self-assemble into micelles through host–guest interactions (Scheme 2). The hydrophobic PHEMA-*g*-(PCL-BM) section assembled as the core, while the hydrophilic CD-*star*-PMAA-*b*-PNIPAM section extended in the shell to afford the micelle solubility in water.

**Fig. 2:**
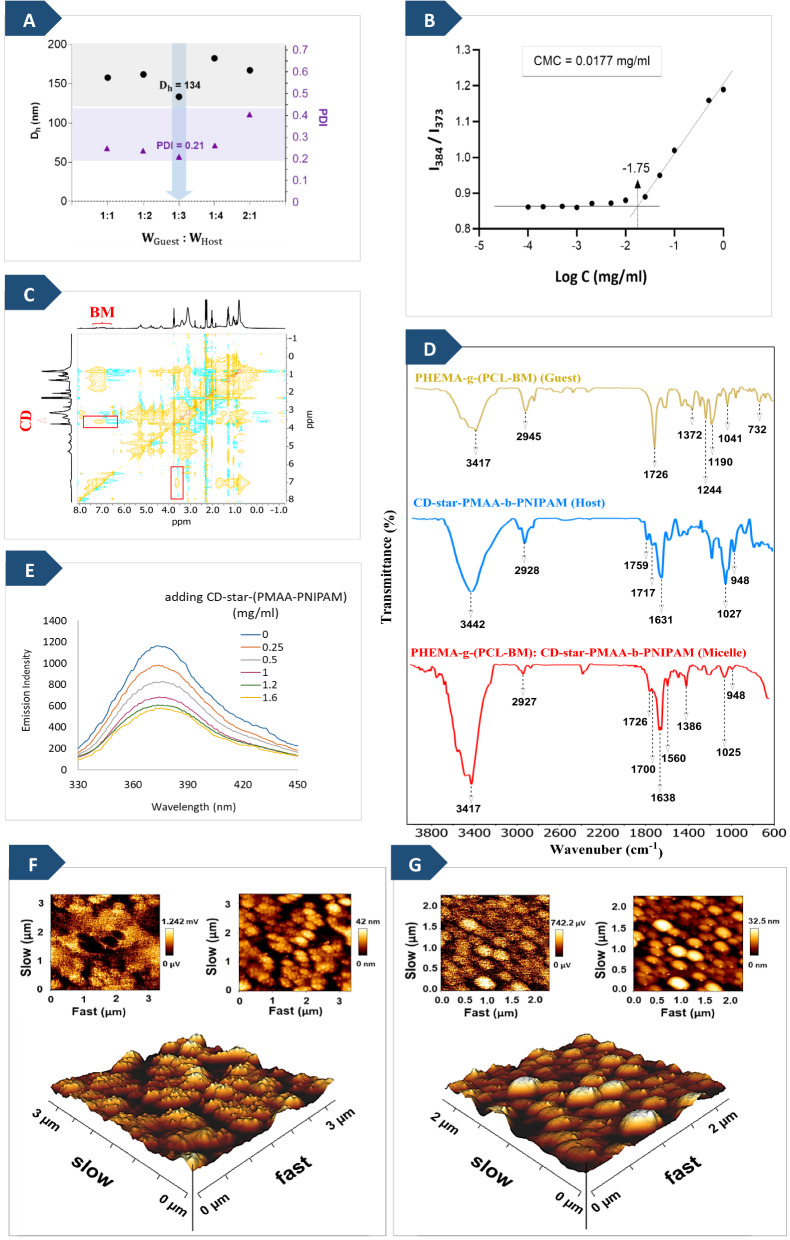
**A** D_h_ and PDI of PHEMA-*g*- (PCL-BM: CD-*star*-PMAA-*b*-PNIPAM) micellar solutions at various weight ratios of BM moieties to β-CD. The ultimate polymer concentration of each sample was set at 0.2 mg/ml. **B** Intensity ratio I_384_/I_373_ from pyrene excitation as a function of the concentration of the PHEMA-*g*-(PCL-BM: CD-*star*-PMAA-*b*-PNIPAM) micelles. **C** 2D-NOESY NMR spectra of the PHEMA-g-(PCL-BM: CD-star-PMAA-b-PNIPAM) inclusion complex in DMSO-d6 as solvent at a guest/host weight ratio of 1:3 (400 MHz). **D** FTIR spectra of PHEMA-g-(PCL-BM) (guest), CD-star-PMAA-b-PNIPAM (Host), and PHEMA-g-(PCL-BM): CD-star-PMAA-b-PNIPAM (micelle). **E** Fluorescence emission spectra of PHEMA-*g*-(PCL-BM) in DMSO/PBS solutions with different CD-*star*-PMAA-*b*-PNIPAM concentrations (λ_ex_ = 297 nm). **F** 3D and 2D atomic force microscopy (AFM) images of PHEMA-*g*-(PCL-BM), **G** PHEMA-*g*-(PCL-BM: CD-*star*-PMAA-*b*-PNIPAM) inclusion complex

Figure 2A shows the DLS results of PHEMA-*g*-(PCL-BM: β-CD-*star*-PMAA-*b-*PNIPAM) micellar solutions at various weight ratios of BM moieties (guest) to β-CD moieties (host). It indicates that the average hydrodynamic diameters (D_h_) of the micelles are 158, 162, 134, 182, and 167 nm and its relative average polydispersity index (PDI) are 0.255, 0.242, 0.210, 0.27, and 0.4 when guest: host weight ratios = 1:1, 1:2, 1:3, 1:4, and 2:1 respectively. The guest/host weight ratio in the range from 1:1 to 1:4 has a low effect on the micelle size and the size distribution. Similar results have been reported in the literature [39, 40]. Based on these results, the weight ratio of PHEMA-*g*-(PCL-BM) to (β-CD-*star*-(PMAA-*b* PNIPAM) in the micelles was fixed at 1:3 in the subsequent measurements, which had a suitable size and size distribution of micelles in water.

The self-assembly of PHEMA-*g*-(PCL-BM: CD-*star*-PMAA-*b*-PNIPAM) was characterized by measuring the CMC value of the blank micelles using the fluorescence spectroscopy method and by employing pyrene as a fluorescent probe at 25 °C. The fluorescence intensity ratio of I_384_/I_373_ versus log C is plotted in Fig. 2B. The I_384_/I_373_ ratio remained almost constant at low graft copolymer concentrations. When the copolymer concentration reached a specific value, the total fluorescence intensity ratio increased remarkably. This is a reflection of the graft copolymer micelles aggregation. The CMC value of PHEMA-*g*-(PCL-BM: CD-*star*-PMAA-*b*-PNIPAM) in an aqueous solution was 0.0177 mg mL^−1^ determined from Fig. 2B. The CMC is an effective parameter characterizing micellar stability, and a lower CMC value indicates relatively higher stability [41].

**Fig. 3:**
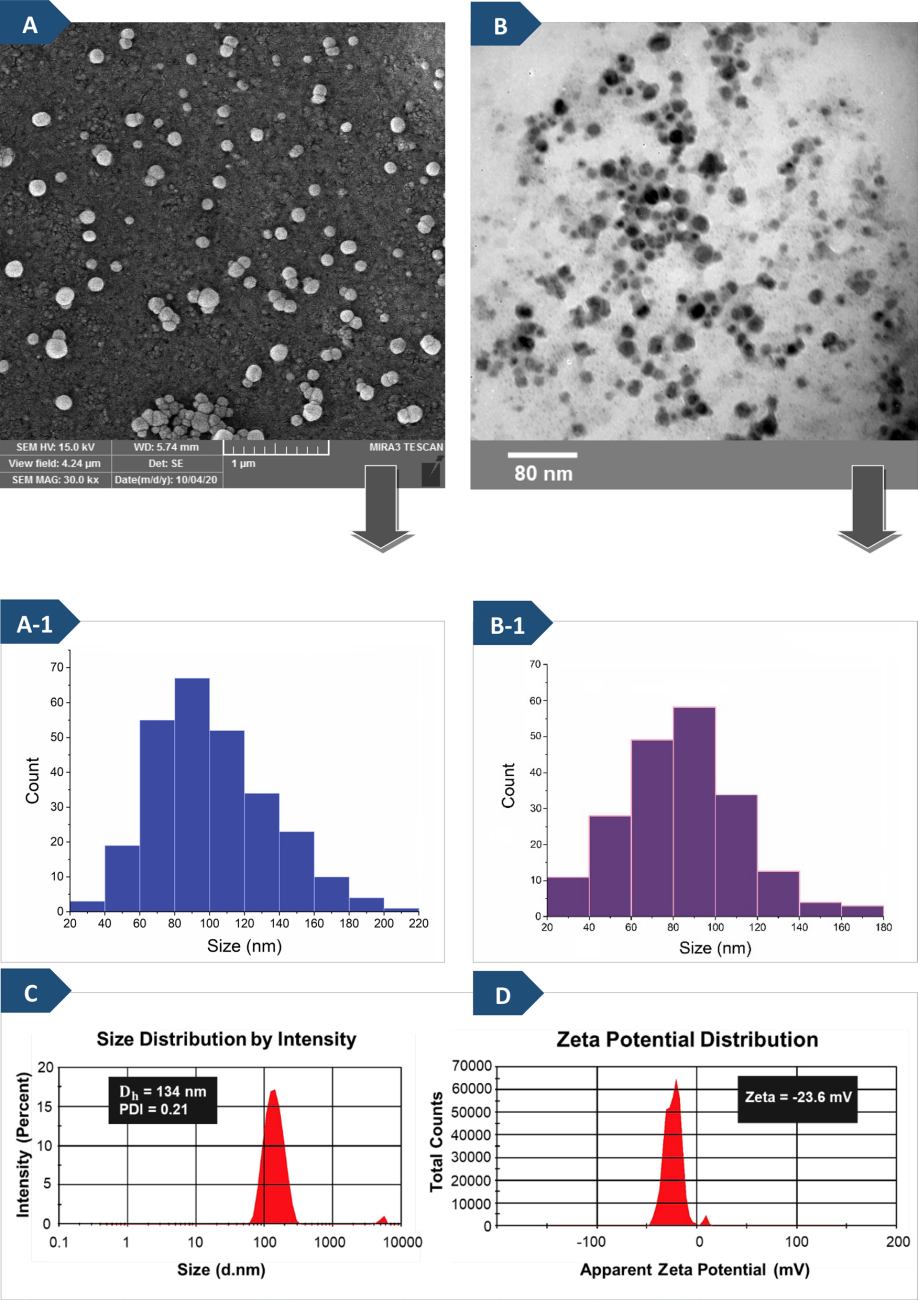
FESEM micrograph (**A**), histogram of particle-size distribution from FESEM (**A-1**), TEM micrograph (**B**), histogram of particle-size distribution from TEM (**B-1**), and hydrodynamic size distribution (**C**) and the zeta potential (**D**) obtained by DLS measurements of synthesized PHEMA-*g*-(PCL-BM: CD-*star*-PMAA-*b*-PNIPAM) micelles in deionized water and 25 ºC (the weight ratio of BM/CD was 1:3)

In this study, the BM moiety of the PHEMA-*g*-(PCL-BM) was fitted into the β-CD cavity of the CD-*star*-PMAA-*b*-PNIPAM via inclusion complexation, forming noncovalent graft copolymer micelles. In order to confirm the inclusion complexation between the host and guest units, the 2D-NOESY NMR spectrum of PHEMA-*g*-(PCL-BM):(CD-*star*-PMAA-*b*-PNIPAM) copolymer micelles was prepared. The cross-peaks in this spectrum identify the protons of guest and host molecules undergoing “through space” dipolar interactions and the formation of inclusion complexes [42–44]. As shown in Fig. 2C, the observed cross-peaks indicate the interaction between BM protons and the inner protons of β-CD in an inclusion complex. The signals appeared at δ 6.8–7.8 ppm [45] attributed to the BM moieties of guest copolymer demonstrate cross-peaks, resulting from dipolar interactions with the signals that occurred at 3.5–3.8 ppm [15, 42, 46] ascribed to the C(h)-H and C(f)-H protons (see Fig. 1 (C-3)) located inside the cavity of β-CD moieties in the host copolymer, which is in accordance with the Yang et al. report [47]. This phenomenon could support the formation of inclusion complexes via host–guest interaction between the BM moieties with β-CD cavities.

In order to demonstrate the successful association of BM units and CD units, the fluorescence of the complexes in the mixed solvent (DMSO/PBS, pH 7.4) was also studied. As shown in Fig. 2E, the PHEMA-*g*-(PCL-BM) concentration was fixed at 0.5 mg/ml, whereas the concentration of CD-*star*-PMAA-*b*-PNIPAM was increased from 0 to 1.6 mg/ml. The pure PHEMA-*g*-(PCL-BM) solution showed typical fluorescence at 378 nm. The increase in CD-*star*-PMAA-*b*-PNIPAM concentration from 0 to 1.2 mg/ml led to a marked decrease in the fluorescence intensity of BM moieties, because a less hydrophobic environment was formed by the association of BM moieties with CD moieties. Therefore, the decrease in the fluorescence intensity of BM moieties upon the addition of CD-*star*-PMAA-*b*-PNIPAM suggests the formation of an inclusion complex between the BM moieties and CD moieties of PHEMA-*g*-(PCL-BM) and CD-*star*-PMAA-*b*-PNIPAM. It is noteworthy that the further increase of the CD-*star*-PMAA-*b*-PNIPAM concentration from 1.2 to 1.6 mg/ml had no obvious influence on the fluorescence intensity of BM moieties. It could be reasonably explained that the BM moieties were almost fully associated with β-CD of CD-*star*-PMAA-*b*-PNIPAM, which similar results reported by some related works [48].

Further investigation into the successful association of BM units and CD units was conducted using FT-IR spectroscopy and AFM as complementary methods. FT-IR spectroscopy is an excellent analytical tool to confirm the formation of inclusion complexes via noncovalent interaction between host and guest molecules by identifying considerable changes in intensity, characteristic peak position, and shape [38, 49, 50]. FTIR spectra were firstly utilized to evidence the formation of graft copolymer micelles via host–guest interactions. The FTIR spectra of the PHEMA-*g*-(PCL-BM) (guest), CD-*star*-PMAA-*b*-PNIPAM (host), and PHEMA-*g*-(PCL-BM: CD-*star*-PMAA-*b*-PNIPAM) (micelle) are shown in Fig. 2D.

Noncovalent interactions between β-CD and BM in the PHEMA-*g*-(PCL-BM: CD-*star*-PMAA-*b*-PNIPAM) micelles causes to minimize the energy of included part of BM and reduce the peak intensity of relevant frequency.

The FTIR spectrum of the guest is characterized by absorption peaks at 1372 cm^−1^ (for C−N stretching vibration), 732 and 1190 cm^−1^ (for C−H vibration), 1244 cm^−1^ (for C−C stretching vibration) [35–37]. All of these peaks are induced by benzimidazole. In the spectrum of the micelle, the absorption bands at 732 and 1244 cm^−1^ disappeared, and the bands at 1041, 1190, and 1372 cm^−1^ aroused from PHEMA-*g*-(PCL-BM) reduced, which is due to the change in the environment of the guest molecule after inclusion in the cavity of CDs. In addition, in the guest spectrum, the bands at 1041 and 1726 cm^−1^ were the C−O−C and C=O vibrations from PHEMA-*g*-PCL, while these peaks decreased in the spectrum of the micelle. Guest and micelle spectra show intense bands at 3417−3480 cm^−1^ for the tertiary nitrogen of benzimidazole ring stretching vibrations. These bands in the guest spectrum and the broad band of host spectrum at 3442 cm^−1^ are merged in the inclusion complex. In the host spectrum, the characteristic peak at 948 cm^−1^ was assigned to the sugar ring skeleton from cyclodextrins in CD-*star*-PMAA-*b*-PNIPAM [51], which was also perceived in the spectrum of graft copolymer micelles. The characteristic stretching vibration of PNIPAM at wavelengths of 1560 and 1638 cm^−1^ remained. These results suggest that the hydrophobic BM moieties inserted into the hydrophobic cavity of β-CD moieties to prepare PHEMA-*g*-(PCL-BM: β-CD-*star*-PMAA-*b*-PNIPAM) micelles via host–guest interaction.

Nuclear magnetic resonance (^1^HNMR) spectroscopy ascertains the inclusion phenomena of the guest molecule inside the β-cyclodextrin cavity as the host molecule [46, 50, 52]. The noncovalent graft copolymer micelles formed in host–guest self-assembling systems were also investigated using the ^1^HNMR method, as shown in section S2.4, supplementary information.

AFM is a suitable non-invasive technique for observing the surface texture of deposited films and measuring the surface roughness at micro-/nano-scale [51, 53]. Both 2D and 3D AFM images of PHEMA-*g*-(PCL-BM) as guest and PHEMA-*g*-(PCL-BM: CD-*star*-PMAA-*b*-PNIPAM) inclusion complex are shown in Fig. 2. The AFM images display nodules on the top surface visible as bright high peaks, while the pores are seen as dark depressions. Visualization of surface topography of PHEMA-*g*-(PCL-BM) (Fig. 2F) and PHEMA-*g*-(PCL-BM: CD-*star*-PMAA-*b*-PNIPAM) inclusion complex (Fig. 2G) by AFM analysis revealed changes in surface morphologies when the including complexes formed between β-CD moieties and BM moieties. The change in the surface topography is proportional to the change in the pore size of the compound and provided strong evidence to confirm the formation of the PHEMA-*g*-(PCL-BM: CD-*star*-PMAA-*b*-PNIPAM) inclusion complex. The average roughness values for PHEMA-*g*-(PCL-BM) and PHEMA-*g*-(PCL-BM: CD-*star*-PMAA-*b*-PNIPAM) inclusion complexes were 9.06 nm and 5.61, respectively. This reveals that PHEMA-*g*-(PCL-BM) has a rough surface, and when BM molecules are embedded in the cavities of β-CD via host–guest interaction, the surface roughness of PHEMA-*g*-(PCL-BM) is changed into a smooth surface. Based on the results of the AFM studies, the inclusion complex obtained by combining the host and guest segments were indeed graft copolymers, indicating that polymerization at each step was successful.

### Morphological characterization

The FESEM micrograph of blank graft copolymer micelles is presented in Fig. 3A. FESEM images of the particle morphology and size distribution of micelles indicate the assemblies are regular spherical structures with an average diameter of around 80 nm.

Furthermore, transmission electron microscopy (TEM) results confirmed the spherical micellar morphology and uniformity of the blank graft copolymer micelles. TEM image of the micelles formed by host–guest interaction with a concentration of 0.2 mg ml^−1^ at 25 ºC is shown in Fig. 3B. The average size observed by the TEM image (~ 80 nm) was in accordance with that measured by SEM (Fig. 3(A-1) and (B-1)).

### Evaluation of size and zeta potential by (DLS) technique

The size and zeta-potential of the micelles are among crucial factors affecting their properties and performances in vivo. The size between 60 and 200 nm is the suitable size of nanocarriers for cancer therapy, and in this size range, is expected to restrict their uptake by the mononuclear phagocyte system and permit for passive targeting of cancerous or inflamed tissues through the enhanced permeation and retention (EPR) effect [54, 55]. The hydrodynamic diameter (D_h_), polydispersity index (PDI), and zeta potential of the micelles were measured via DLS, as shown in Fig. 3C, D. It was found that the D_h_ of the micelles was about 134 nm at 25 °C and the PDI value was about 0.216, which shows a narrow size distribution. The present observation showed that the average size of micelles measured by DLS differed from those obtained from TEM and SEM analyses. This is mainly due to the micelles were swollen in water in the DLS analysis, and the hydrated diameter of micelles in the liquid is determined [45, 48]. As seen in Fig. 3D, the graft copolymer micelles revealed a high negative zeta potential value (− 23.6 mV) due to the presence of many carboxylate groups of the PMAA segment in polymer chains bonded onto the surface of micelles, which is in accordance with the Yang et al. report [56].

### Thermo-response of the noncovalent graft copolymer micelles

The main aim of incorporating NIPAM units within these amphiphilic graft copolymers was to bring about a thermo-sensitive behavior to prepared micelles. The polymers containing PNIPAM indicate lower critical solution temperature (LCST) behavior, which corresponds to a thermo-sensitive hydrogen bonding ability of amide groups with the water solvent. LCST of PNIPAM is altered through the attachment of PMAA chains, and it is expected to be lowered if the PMAA block is hydrophobic (at low pH) and raised if the PMAA block is hydrophilic (at high pH) [57]. The temperature-responsive behavior of the graft copolymer micelles (with a host to guest ratio of 3 to 1 (w/w)) was investigated by turbidimetry and DLS measurements at various temperatures (from 30 to 50 °C).

**Fig. 4:**
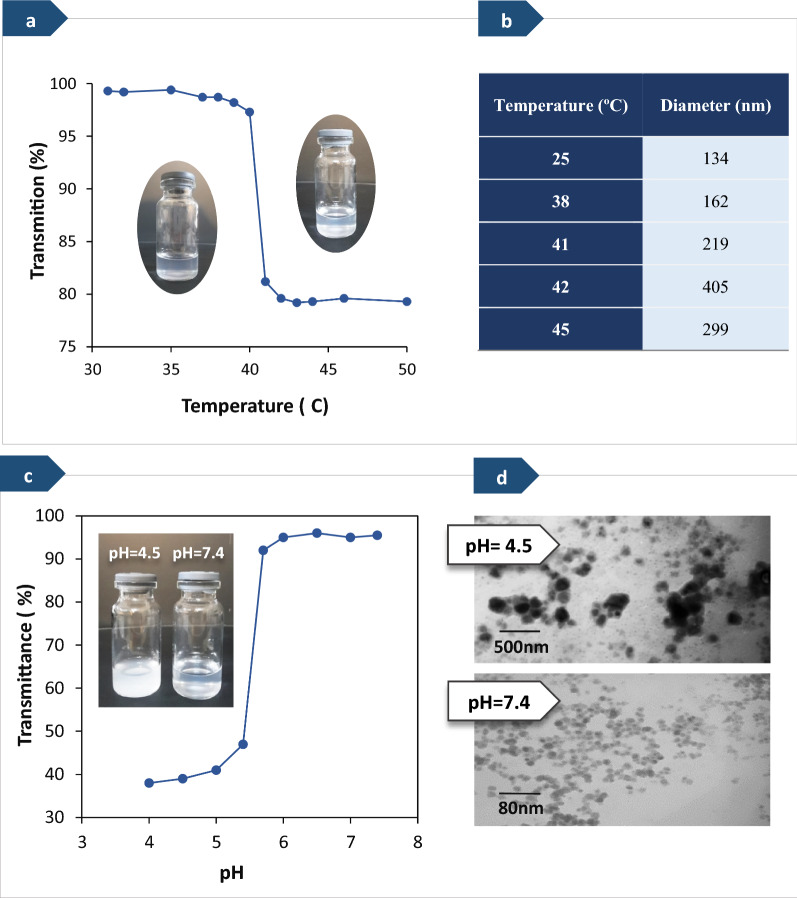
**a** A visual illustration of the PHEMA-*g*-(PCL-BM: β-CD-*star*-PMAA-*b*-PNIPAM) micelles in aqueous solution and LCST determination by the UV–Vis spectrum and **b** diameter change of the micelles versus temperature (0.2 mg mL^−1^). **c** pH-dependence transmittance at the wavelength of 600 nm for fabricated polymeric micelles in water at ambient temperature and **d** TEM images of the graft copolymer micelles at pH = 4 and 7.4 at 25 °C (0.2 mg mL^−1^).

As the first step, visual observations were carried out to study the temperature-responsive behavior of PHEMA-*g*-(PCL-BM: CD-*star*-PMAA-*b*-PNIPAM) graft copolymer micelles in water. The results obtained from visual observation are shown in Fig. 4a, b). These results showed that the micellar solution was transparent below 40 °C, while turbid above 40 °C. In the next step, turbidity variation of the samples at various temperatures was measured using a UV–vis spectrophotometer at a wavelength of 600 nm, and the results are shown in Fig. 4a. In this way, the LCST was determined from the transmittance of samples containing polymeric micelles as a function of temperature, as depicted in Fig. 4a. Below the LCST, the PNIPAM block was hydrophilic and water-soluble, owing to the formation of hydrogen bonds between the water and amide groups of PNIPAM, and the transmittance percentage was approximately 100%. When the temperature reached above the LCST, the hydrophobic interactions became dominant, and the transmittance reduced since the PNIPAM blocks became hydrophobic and started to aggregate in the aqueous solution. It is worth noting that the LCST value of the graft copolymer PHEMA-*g*-(PCL-BM: β-CD-*star*-PMAA-*b*-PNIPAM) micelles was pH-dependent. The incorporation of hydrophilic PMAA block into PNIPAM block increased the LCST of the copolymer and decreased the phase transition rate by stabilizing polymer dissolution [58]. In this work, the same phenomenon was observed.

On the other hand, incorporating hydrophobic guest moieties into the polar cavities of β-CD as host moieties prevents further increase of LCST [17]. As shown in Fig. 4a, the transmittance of the graft copolymer micelles reduced from 100% to almost 80% with the enhancement of temperatures from 31 to 50 °C in neutral pH, being in accordance with the previously reported studies [31, 57, 59]. The LCST of the graft copolymer PHEMA-*g*-(PCL-BM: β-CD-*star*-PMAA-*b*-PNIPAM) micelles was found to be about 40−410 °C.

Further investigation into the thermoresponsive behavior of micelles was conducted using DLS measurements as a complementary method. The results obtained by DLS at various temperatures are presented in Fig. 4b. When the temperature of the sample solution was reached its LCST value, the association of aggregates occurred, and the diameter of aggregates significantly increased from 134 to 405 nm as the temperature increased from 25 to 410 °C. A further increase in temperature caused a slight reduction in the diameter of the aggregates (450 °C), presumably because the interactions between hydrophobic groups became dominant and further heating the solutions triggered the shrinking of the aggregates as a result of dehydration [60, 61].

### pH-Response of graft copolymer micelles

The environment of tumor tissues is different from that of the normal tissues in many aspects, including high intracellular adenosine triphosphate (ATP) concentration, a low pH value, and overexpression of some biological enzymes, which is the basis for the design of intelligent drug carriers [62, 63].

The pH-responsive noncovalent graft copolymers can be defined as polyelectrolytes that include in their backbone or side chains weak acidic (e.g., carboxylic and sulfonic acids) or basic groups (e.g., amines, imidazole, and pyridine) that either accept or donate H^+^ ions in response to environmental pH changes [64]. Our results showed that micelles formed from PHEMA-*g*-(PCL-BM: β-CD-*star*-PMAA-*b*-PNIPAM) have a pH-responsive behavior due to the presence of benzimidazole units and PMAA units on the graft copolymer chains.

Under acidic conditions, host–guest interactions between BM segments and β-CD segments can be cleaved due to the BM could be protonated in the acidic environment and causes dissociation of inclusion complexes [47, 65]. On the other hand, at lower pH values, carboxyl groups (−COOH) of the PMAA moieties are protonated, and hydrophobic interactions dominate, leading to reduction of the volume of the polymer containing the carboxyl groups. With increasing the pH, −COOH groups start to deprotonate, resulting in a high charge density in the polymer. Due to Coulomb repulsion, the negative charge of carboxylate ions would induce repulsive forces and increase the free volume [58]. The pH at which these changes occur is called “transition pH”, which depends on the pK_a_ value of the polymer [66].

The pH-responsive behavior of the micelles from host–guest mixtures in deferent buffer solutions was investigated through UV–Vis transmittance at a wavelength of 600 nm and transmission electron microscopy (TEM). Figure 4c demonstrates transmittance changes of the polymeric micelles with changes in pH. It can be seen that the micelles displayed a significant increase in transmittance at ca. pH 5.5, which was very near to the pKa of PMAA units (about 5.6 [67, 68]). At pH below the pK_a_ value (5.5), PMAA exhibits a marked pH-induced conformational transition, and owing to the formation of aggregates of the hydrophobic segments after the disassociation of the inclusion complexes, the transmittance had relatively lower values, being in accordance with the previously reported studies [68, 69].

The effect of pH on the size of the graft copolymer micelles was investigated through TEM. Based on TEM analysis, it was found that the blank graft copolymer micelles had a nanosized spherical shape with a size of approximately 80 nm at pH 7.4, while the particle size demonstrated a significant increase at pH 4.5, as shown in Fig. 4d.

#### Loading of DOX in noncovalent graft copolymer micelles

Generally, there are three primary methods for loading drugs into polymer micelle cores, (1) physical entrapment or solubilization, (2) polyionic complexation, and (3) chemical conjugation [25]. Although the covalent conjugation strategies have some limitations for loading the drugs due to the chemical modification of drugs’ structure, which may cause drugs to be less effective, the physical encapsulation via hydrophobic interactions or charge does not lead to any change in the chemical structure of the drug [70]. The electrostatic interactions between the drug and the polymeric matrix are one of the most important mechanisms in the physical encapsulation method. Additionally, hydrogen bonding could also act as the main force between drugs and carriers [70, 71]. DOX is a potent chemotherapeutic antineoplastic agent, but it has a short in vivo biological half-life and seriously injured the healthy cells when killing the cancerous cells [72]. In this research, to verify the feasibility of using the self-assembled micelles based on graft copolymer as a 'smart' drug carrier in cancer treatment, DOX.HCl was selected as a model hydrophilic drug and loaded into the fabricated micelles by a dialysis method [33, 48, 45]. Although DOX.HCl displays water solubility character, it keeps the identical physicochemical and biological properties as the hydrophobic form of DOX, while it still has a main hydrophobic structure [70].

**Fig. 5:**
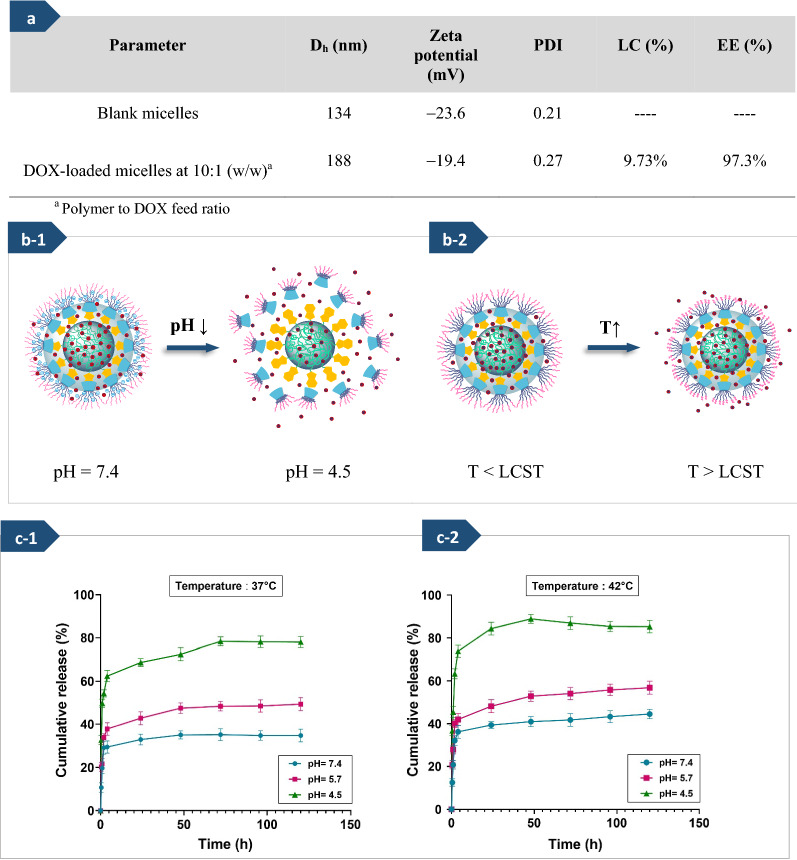
Hydrodynamic diameter (D_h_), zeta potential, and size distribution (PDI) of blank micelles and DOX-loaded micelles, along with drug-loading content (LC%) and encapsulation efficiency (EE%) of micelles (**a**). The schematic illustration of drug release process from PHEMA-*g*- (PCL-BM: β-CD-*star*-PMAA-*b*-PNIPAM) micelles ((**b-1**), (**b-2**)), in vitro DOX release profiles of PHEMA-g- (PCL-BM: β-CD-star-PMAA-b-PNIPAM) micelles at three pH values (7.4, 5.7, and 4.5), and two temperature values (37 (**c-1**), and 42 °C (**c-2**))

The D_h_, polydispersity index, and zeta potential of DOX-loaded micelles were measured through DLS, as summarized in Fig. 5a. The particle size of DOX-loaded micelles indicated that encapsulation of DOX into micelles increased the diameters of micelles, which similar results reported by some related works [73, 74]. The pK_a_ of DOX is 8.3, so it has positively charged at pH ≤ pK_a_ due to the existence of the amino groups. The amine functional groups (−NH_2_) in the DOX chemical structure can absorb the protons and convert them to the cationic form (−NH_3_^+^) [70]. Thus, it was attracted to the negatively charged micelles and effectively loaded into the core of the micelles based on the hydrophobic and electrostatic interactions between the drug molecules and the polymer chains [70, 75]. Furthermore, DOX could also be entrapped into polymeric micelles architecture through hydrogen bonding between the side chains of graft copolymer and DOX. Therefore, after DOX loading on micelles, owing to the presence of electrostatic interaction between carboxylate groups of the PMAA segment in polymer chains bonded onto the surface of micelles and DOX with positive charge [56, 76], the zeta potential of the drug-loaded micelles slightly reduced from − 23 to − 19 mV. The LC and EE percentages of DOX in the graft copolymer micelles (polymer to drug ratio of 10 to 1) were 9.73% and 97.3%, respectively. These high loading and encapsulation efficiency may be related to the hydrophobic PCL blocks in micelles core and the hydrophobic cavities of β-CD, which enable them to load a large amount of drugs [45, 75]. Therefore, the assembled micelles based on the graft copolymer (PHEMA-*g*-(PCL-BM: β-CD-*star*-PMAA-*b*-PNIPAM) can suggest promising features for their use as controlled drugs carriers.

#### Release of DOX from noncovalent graft copolymer micelles

The major subject for designing an effective drug carrier is that it can maintain its stability under normal physiological conditions (pH ~ 7.4) but selectively release drug molecules by sensing pH decrease in the vicinity of cancer tissue (pH ≤ 5) and/or the endosome-lysosome (pH ~ 4.5) [77]. To evaluate the process of DOX release behavior of the fabricated PHEMA-*g*-(PCL-BM: β-CD-*star*-PMAA-*b*-PNIPAM) micelles, two temperature values (37 and 42 °C) and three different pH values (7.4, 5.7, and 4.5), were chosen to emulate the normal physiological and cancerous tissue conditions. As seen in Fig. 5(c-1) and (c-2), under the physiological conditions (pH 7.4), the DOX-releasing efficiencies remained relatively stable, and only about 32 and 35% of the DOX were released within 24 h at 37 and 42 °C, respectively. In contrast, under cancerous conditions (pH 5.7 and 4.5), the DOX release was significantly accelerated, especially at pH 4.5. At a pH value of 4.5, the BM molecules were protonated, which led to the dissociation of host–guest inclusion complexes between BM segments and cyclodextrin segments [17, 48, 69]; approximately 70 and 85% of the loaded DOX were released from fabricated micelles in the first 24 h at 37 and 42 °C, respectively.

On the other hand, the fast drug release at a temperature above the LCST of micelles was due to the that the collapse of PNIPAM segments located in the shell increased the spatial distance between polymer chains and shorten release way [41]. The present observation showed that sustained release is carried out by the departure of cyclodextrins and contraction of temperature-sensitive segments, as shown in Fig. 5(b-1) and (b-2).

All results proved that the designed nanocarrier based on the host–guest inclusion complex was dual-responsive and should be suitable for cancerous drug delivery systems.

### In vitro biocompatibility evaluation and cytotoxicity assay

**Fig. 6:**
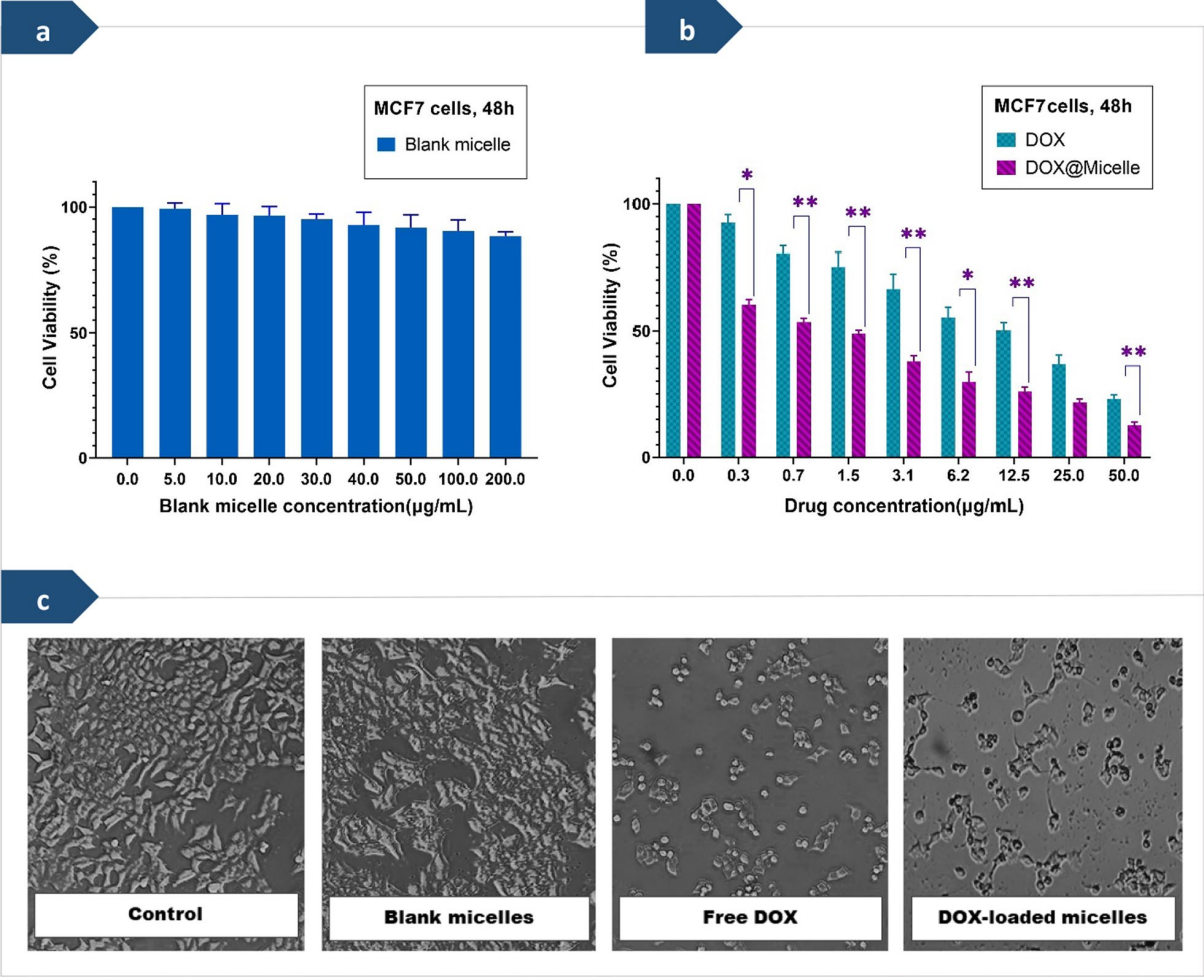
**a** Cell viability of MCF-7 cells after treatment with different concentrations of the blank micelles (PHEMA-*g*- (PCL-BM: β-CD-*star*-PMAA-*b*-PNIPAM)). **b** Cell viability versus drug dose of the free doxorubicin (DOX), and the drug-loaded micelles against human breast cancer cells (MCF-7) after incubation for 48 h. Each value represents the mean value ± standard deviation (n = 3, *p < 0.05, ** p < 0.01, *** p < 0.001). **c** Morphological changes of the MCF-7 cancer cell line after treatment with various formulations for 48 h (blank micelles concentration = 200 μg/ml and free DOX & DOX-loaded micelles concentrations = 50 μg/ml).

The biocompatibility of the prepared micelles, as well as the cytotoxicity of DOX-loaded micelles, were investigated by the colorimetric MTT assay against MCF-7 cells in the time period of 48 h, and the obtained results were compared with the cytotoxicity effect of free DOX as the reference, as shown in Fig. 6. It was observed that the graft copolymer micelles alone had much low cytotoxicity on MCF-7 cell lines (Fig. 6a). The determined half-maximal inhibitory concentration (IC50) values of free DOX and DOX-loaded polymeric micelles against MCF-7 cells were ca. 18.29, and 1.75 μg/ml, respectively. As can be seen in Fig. 6b, compared with blank polymeric micelles, the viability of the cells treated with DOX-loaded polymeric micelles at a very low dose of drug exhibited a rapid decrease. The cell viability rate of DOX-loaded polymeric micelles at the drug dose of 50 μg/ml was almost 12.7%, while the cell viability rate of free DOX at the same drug dose was only 23.1%. This observation displayed that the cell growth inhibition of DOX-loaded polymeric micelles was higher than that of free DOX at the same drug dose. The higher tumor growth inhibition efficacy observed for the DOX-loaded polymeric micelles can be attributed to the improved tumor accumulation of DOX in the MCF-7 cells and fast intracellular drug release [45]. The cells treated with blank micelles compared with untreated cells demonstrated no significant morphological changes and revealed their evenly and intact shapes.

We further investigated the viability and morphological changes of the MCF-7 cells after treatment with various formulations for 48 h, as shown in Fig. 6c. MCF‑7 breast cancer cells have a shuttle, triangle, or irregular shape, and they have a habit of clumping into large aggregates [78]. Under microscopic observation, compared with the control group, the tumor cells in blank micelles-treated groups had almost no change, but DOX treatment destroyed the compact structure and shuttle morphology of tumor cells. These results were entirely consistent with the viability of the MCF-7 cells after treatment with different doses of the drug-free micelles. In contrast, in the DOX-loaded micelles-treated group, the structure of tumor cells was severely disrupted and many cells separated from tumor cells, suggesting that the drug was endowed with strong infiltration capability and effective antitumor depending on the micelle delivery system. These results implied that DOX was efficiently delivered into MCF-7 cells by PHEMA-*g*- (PCL-BM: β-CD-*star*-PMAA-*b*-PNIPAM) micelles, which corroborates with the MTT results in this study.

### Morphological assessment of apoptotic cells using DAPI staining

**Fig. 7:**
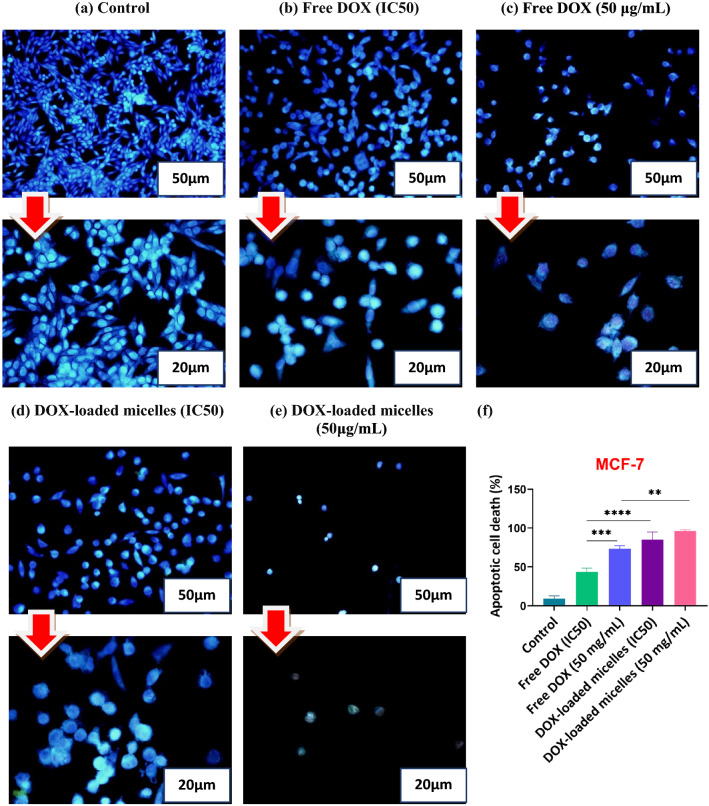
Fluorescence microscopic images of DAPI stained MCF-7 cells following 48 h of exposure to untreated cells (control) (**a**), free DOX (IC50) (**b**), free DOX (50 μg/mL) (**c**), DOX-loaded micelles (IC50) (**d**), and DOX-loaded micelles (50 μg/mL) (**e**). The percentages of apoptotic cell death in MCF-7 cells after being exposed to free DOX, and DOX-loaded micelles (**f**). As displayed in the diagram, DOX-loaded polymeric micelles yield highly considerable apoptosis (p < 0.01) in comparison to free DOX. Each value represents the mean value ± standard deviation (n = 3, *p < 0.05, ** p < 0.01, *** p < 0.001, ****p < 0.0001). Images were taken at magnification (×200) and (×400)

The 4,6-diamidino-2-phenylindole (DAPI) is a cell-permeable fluorescent compound that binds to fragmentation of DNA and condensation of chromatin as changes in the nucleus [76]. In this regard, DAPI staining was utilized as a complementary assay to assess the morphological alterations induced by apoptosis in MCF-7 cells. MCF-7 cells were treated with free DOX and drug-loaded PHEMA-*g*- (PCL-BM: β-CD-*star*-PMAA-*b*-PNIPAM) micelles with two concentrations (in the range of IC50 values and drug dose of 50 μg/ml). The morphological changes in cell nuclei after 48 h treatment were observed by fluorescence microscopy, and the results are shown in Fig. 7. The images of cells treated with free DOX and DOX-loaded PHEMA-*g*- (PCL-BM: β-CD-*star*-PMAA-*b*-PNIPAM) micelles demonstrated the signs of apoptosis such as cell shrinkage, nuclear fragmentation, loss of cell–cell contact, and chromatin condensation. In comparing these groups, the MCF-7 cells treated with DOX-loaded micelles revealed high-level apoptosis evidence in contrast to the free DOX, but no apoptotic cells were detected in the control group.

## Conclusions

In this study, novel temperature and pH double stimuli-responsive amphiphilic graft copolymer micelles based on benzimidazole-terminated PHEMA-*g*-PCL as guest polymer and β-CD-*star* (PMAA-*b*-PNIPAM) as host polymer were designed and synthesized exploiting the host–guest interaction between benzimidazole (BM) and β-cyclodextrin (β-CD) moieties. The fabricated graft copolymer had a brush-like structure. The advantages of such micellar systems based on graft copolymers include their great self-assembly ability in an aqueous environment, low CMC value, rapid pH/thermo-responsiveness, and desired loading capacity, as well as the effective release of the drug. The fabricated micelles were utilized as thermo/pH dual-responsive nanocarriers that demonstrated the LCST between 40 and 41 °C. Doxorubicin hydrochloride was effectively loaded into the graft copolymer micelles as a model drug. The in vitro drug release evaluation demonstrated that the temperature and acidic conditions facilitated more the release of the model drug so that the high temperature and acidic pH of cancer cells promoted the release of the drug from the smart nanocarrier. In vitro cell cytotoxicity evaluation exhibited good biocompatibility of the blank micelles based on graft copolymers. Moreover, the results obtained from the cytotoxicity analysis of the fabricated copolymer micelles displayed that DOX-loaded micelles exhibited higher anticancer effects as compared to free DOX against MCF-7 cell line. In addition, DAPI staining results proved the successful induction of apoptosis in cancerous cells by drug-loaded micelles. Therefore, PHEMA-*g*-(PCL-BM: β-CD-*star*-PMAA-*b*-PNIPAM) noncovalent graft copolymer micelles appear promising candidates for intelligent drug delivery systems.

## Supplementary Information

**Additional file 1.** Additional characterization data, calibration curves.

## Data Availability

All data generated or analyzed during this study are included in this published article and its additional files.
