# Supplementary material for: Thermo/pH dual-responsive micelles based on the host–guest interaction between benzimidazole-terminated graft copolymer and β-cyclodextrin-functionalized star block copolymer for smart drug delivery
Source: J Nanobiotechnology. 2022 Feb 22;20:91. doi: 10.1186/s12951-022-01290-3 (PMC8864802; doi:10.1186/s12951-022-01290-3)
Supplement: Supplementary file 1 — Additional file 1. Additional characterization data, calibration curves. [file 12951_2022_1290_MOESM1_ESM.docx]

*Supplementary information*

**Thermo/pH dual-responsive micelles based on the host–guest interaction between benzimidazole-terminated graft copolymer and β-cyclodextrin-functionalized star block copolymer for smart drug delivery**

Floria Adeli^a^, Farhang Abbasi^b^, Mirzaagha Babazadeh^a^, Soodabeh Davaran^c^

^a^ Department of Chemistry, Tabriz Branch, Islamic Azad University, Tabriz, Iran

^b^ Institute of Polymeric Materials (IPM), Sahand University of Technology, Tabriz, Iran

^c^ Research Center for Pharmaceutical Nanotechnology, Faculty of Pharmacy, Tabriz University of Medical Sciences, Tabriz, Iran

**Correspondence:** Prof. Farhang Abbasi, Institute of Polymeric Materials (IPM), Sahand University of Technology, Tabriz, Iran.

e-mail: [f.abbasi@sut.ac.ir](mailto:f.abbasi@sut.ac.ir)

Tel/Fax: +98-41-33459090

**S1. Experimental Section**

The calibration curves of DOX in phosphate buffer saline (pH 7.4, 5.7, 4.5) were constructed by plotting the absorbance value versus the concentration of DOX. (Figure S1).

**
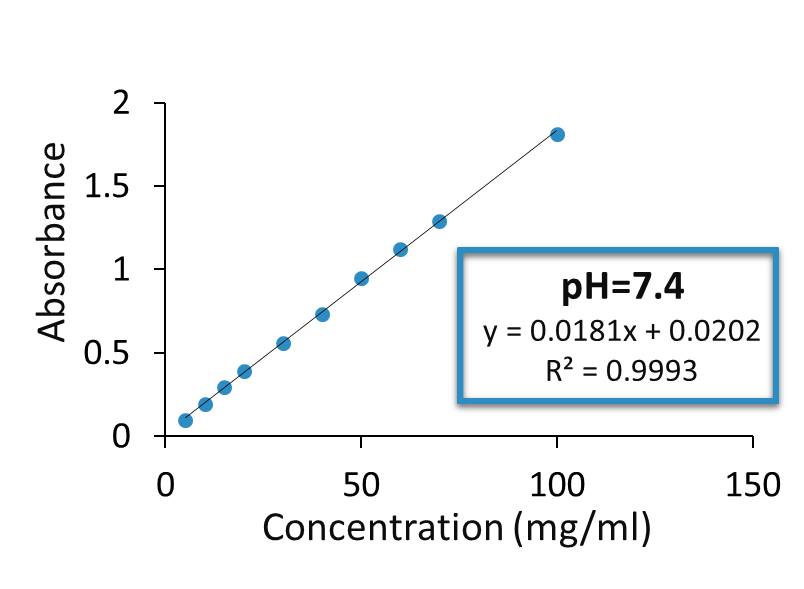
**

**
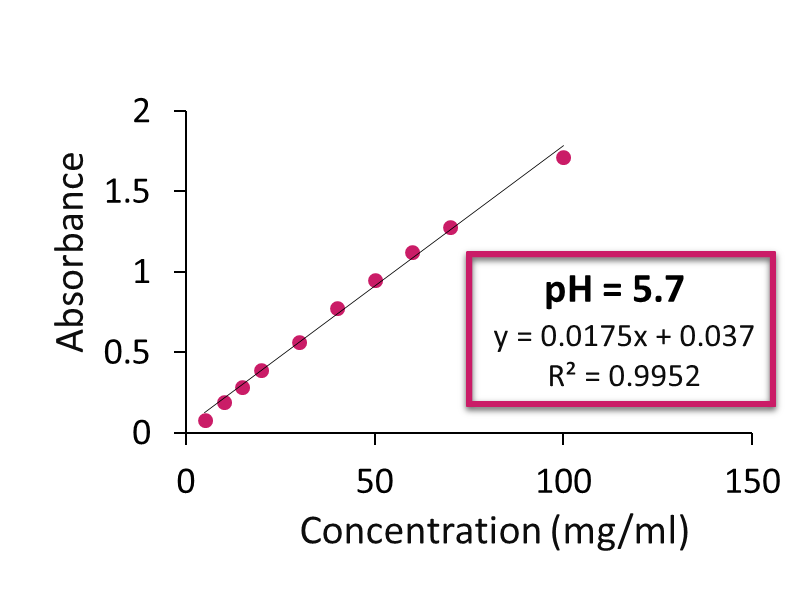
**

**
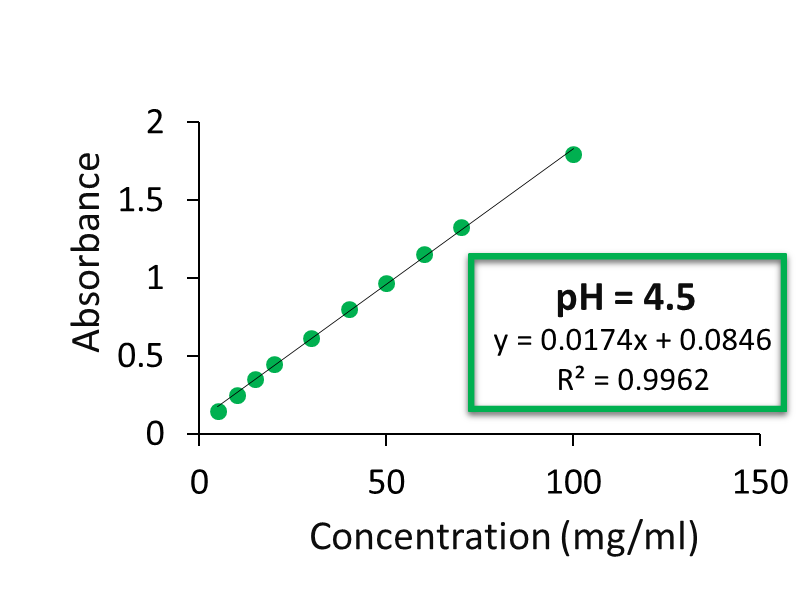
**

**Figure S1.** Calibration curves of Dox in PBS (pH 7.4, 5.7, and 4.5). Absorbance for different concentrations of DOX was measured using UV-Vis spectrophotometer at wavelength of 480 to acquire a linear correlation.

**S2. Results and discussion Section**

**S2.1. Characterization of guest polymers**

The ATR-FTIR spectrum of the PHEMA showed the characteristic absorption bands due to the stretching vibration of C=O group at 1720 cm^−1^, stretching vibration of C–O group at 1388 cm^−1^, C–H bending vibration at 1451 cm^−1^, aliphatic C–H stretching vibrations at 2883 and 2944 cm^−1^, and C–O–C stretching vibration at 1273 cm^−1^ (1). Furthermore, the broad and strong band centered at 3400 cm^−1^ was attributed to the O–H group of the PHEMA (Fig. S2(1)) (2). The existence of PCL segments in PHEMA-*g*-PCL could be conﬁrmed by IR spectrum. According to Fig. S2(2), the intensive carbonyl bond absorption peak at 1726 cm^−1^ should be assigned to the carbonyl bond of PCL segments and the broad absorption at 3419 cm^−1^ was ascribed to the absorption band of the hydroxyl group in PHEMA-*g*-PCl (2). In the spectrum of PHEMA-g-(PCL-BM), the peaks at 1726 and 1041 cm^−1^ were the C=O and C−O−C vibration corresponding to the both PHEMA and PCL segments (Fig. S2(3)). The main absorption bands in this sample induced by benzimidazole could be listed as: stretching vibration of C_arom_−N_benzimi_ group at 1372 cm^−1^, stretching vibrations of C_aliph_−N_benzimi_ at 3417–3480 cm^−1^, stretching vibration of C−C at 1244 cm^−1^, and C−H vibration at 732 and 1190 cm^−1^(3-5).

**Figure S2.** The FTIR spectra of PHEMA (1), PHEMA-g-PCL (2), and PHEMA-g-(PCL-BM) (3)

**S2.2. Characterization of host polymers**

The ATR-FTIR spectrum of the PMAA showed the characteristic absorption bands due to C=O stretching vibration of carboxylic group at 1722 cm^−1^, and 2929 cm^−1^ due to CH_2_ stretching (Fig. S3(1))(6).

In the FTIR spectrum of PMAA−*b*−PNIPAM, the peaks at 1700, 1640, and 3442 cm^−1^ were related to the stretching vibration of carbonyl group, amid carbonyl group, and −NH secondary amid, respectively (Fig. S3(2))(7, 8).

As shown in Fig. S3(3), the new peak at 948 cm^−1^ was assigned to the characteristic peak of α-pyran vibration from β−CD structure (9). On comparing with the spectrum of PMAA-*b*-PNIPAM, the appearance of new peak at 1027 cm^−1^ in β-CD-*star*-(PMAA−*b*−PNIPAM) was assigned to the involved skeletal vibration of C−O stretching (10). These results confirmed that that β−CD−*star*-(PMAA−b−PNIPAM) was successfully synthesized.

**Figure S3.** The FTIR spectra of PMAA (1), PMAA-*b*-PNIPAM (2), and β-CD-*star*- (PMAA-*b*-PNIPAM) (3).

**S2.3. Calculation of** $\bar{\mathbf{DP}}$**_n_ and** $\bar{\mathbf{M}}$**_n, NMR_ from the NMR results**

**S2.3(a).**

The degree of polymerization ($\bar{\mathrm{DP}}$_n_) and number average molecular weight ($\bar{M}$_n_) of the synthesized PHEMA were calculated from the ^1^H NMR data using the following equations (11).

${\bar{\mathrm{DP}}}_{n‚PHEMA}= \frac{I_{H(polymer)}}{I_{H\left( end-group \right)}}= \frac{\frac{I_{(-CH2OH)}}{2}}{\frac{I_{(ph)}}{5}}= \frac{5 10․86}{2 O․79}\simeq69$ (1)

$\bar{M}$_n, PHEMA_ = ($\bar{\mathrm{DP}}$_n, PHEMA_ × MW _HEMA_) + MW _RAFT agent_

$=\left( 69 130․14 \right)+279․38\simeq$9260 g/mol (2)

**S2.3(b).**

The number average molecular weight of the synthesized PHEMA-*g*-PCL was calculated from the ^1^H NMR data using the following equation (12).

$\bar{M}$_n, PHEMA-_*_g_*_-PCL_ = ($\bar{\mathrm{DP}}$_n, PCl_ × 114.14 + MW _HEMA_) × $\bar{\mathrm{DP}}$_n, PHEMA_

= (20 × 114.14 + 130.14) × 69 $\simeq$ 166500 g/mol (3)

where $\bar{\mathrm{DP}}$_n, PCl_ is the DP of PCL in the copolymer.

$\bar{\mathrm{DP}}$_n, PCl_ = $\frac{I_{H (peak d)}}{I_{H(\mathrm{peak}d^{'})}}$ = $\frac{1}{0․05}=$ 20

**S2.3(c).**

The Coupling efficiency percentage and the number average molecular weight of the synthesized PHEMA-*g*-(PCL-BM) were calculated using the following equations (13).

Coupling efficiency (%)= $\frac{I_{(-\mathrm{CH}_{2}\mathrm{CH}_{2}-N_{\mathrm{benzimi}})}}{I_{(-\mathrm{CH}_{2}OCO-)}}$ = $\frac{1․69}{2․39}\simeq$ 70% (4)

$\bar{M}$_n, PHEMA-_*_g_*_-(PCL-BM)_ = $\bar{M}$_n, PHEMA-_*_g_*_-PCL_ + MW_BM_ × coupling efficiency × $\bar{\mathrm{DP}}$_n, PHEMA_

= 166500 + (118.14 × $\frac{70}{100} 69)\simeq$ 172200 g/mol (5)

**S2.3(d).**

The degree of polymerization and number average molecular weight of the synthesized PMAA were calculated from the ^1^H NMR data using the following equations.

${\bar{\mathrm{DP}}}_{n‚PMAA}= \frac{I_{H(polymer)}}{I_{H\left( end-group \right)}}= \frac{I_{(-\mathrm{CH}_{2}-)}/2}{I_{(ph)}/5}= \frac{5 4․48}{2 o․55}\simeq$ 21 (6)

$\bar{M}$_n, PMAA_ = ($\bar{\mathrm{DP}}$_n, PMAA_ × MW _MAA_) + MW _RAFT agent_

= (21 × 86) + 279.38 $\simeq$ 2085 g/mol (7)

**S2.3(e).**

The number average molecular weight of the synthesized PMAA-*b*-PNIPAM was calculated from the ^1^H NMR data using the following equation.

$${\bar{\mathrm{DP}}}_{n‚PNIPAM}= \frac{I_{H\left( \mathrm{PNIPAM} \right)}}{I_{H\left( \mathrm{PMAA} \right)}}\times{\bar{\mathrm{DP}}}_{n‚PMAA}= \frac{I_{H\left( c \right)}}{\frac{I_{H\left( a \right)}}{2}-I_{H\left( c \right)}}\times{\bar{\mathrm{DP}}}_{n‚PMAA}$$

$=$ $\frac{1․97}{\frac{6․72}{2} - 1․97}$ $21\simeq30$ (8)

$\bar{M}$_n, PNIPAM_ = $\bar{\mathrm{DP}}$_n, PNIPAM_ × MW _NIPAM_ $=\left( 30 113.14 \right)\simeq$ 3394 g/mol (9)

$\bar{M}$_n, PMAA-PNIPAM_ = $\bar{M}$_n, PNIPAM_ + $\bar{M}$_n, PMAA_ = 3394 + 2085 $\simeq$ 5480 g/mol (10)

**S2.3(f).**

The number average molecular weight of the synthesized CD-*star*-PMAA-*b*-PNIPAM was calculated from the ^1^H NMR data using the following equation.

$\bar{M}$_n, NMR_ = $\bar{M}$_n,_ _PMAA-b-PNIPAM_ × 7 + 1138.98 $\simeq$ 39500 g/mol (11)

Here, 7 is the number of the PMAA-*b*-PNIPAM chains on each β-CD core and 1138.98 g/mol is the molecular weight of β-CD.

**S2.4. Characterization of noncovalent graft copolymer micelles**

In this study, formation of noncovalent graft copolymer micelles was also confirmed by ^1^HNMR technique. Chemical shifts and the changes of the chemical shifts of protons in BM: CD complex compared with the pure compounds of BM and CDs (Figure S4).

**Figure S4.** The ^1^HNMR spectra of guest (1), host (2), and micelle (3) in DMSO-d6 at 25ºC

**References**

1. Davaran S, Ghamkhari A, Alizadeh E, Massoumi B, Jaymand M. Novel dual stimuli-responsive ABC triblock copolymer: RAFT synthesis,“schizophrenic” micellization, and its performance as an anticancer drug delivery nanosystem. Journal of colloid and interface science. 2017;488:282-93.

2. Yuan W, Yuan J, Zhang F, Xie X, Pan C. Synthesis, characterization, crystalline morphologies, and hydrophilicity of brush copolymers with double crystallizable side chains. Macromolecules. 2007;40(25):9094-102.

3. Gao Y, Li G, Zhou Z, Guo L, Liu X. Supramolecular assembly of poly (β-cyclodextrin) block copolymer and benzimidazole-poly (ε-caprolactone) based on host-guest recognition for drug delivery. Colloids and Surfaces B: Biointerfaces. 2017;160:364-71.

4. Haque RA, Iqbal MA, Ahamed MBK, Majid AA, Hameed ZAA. Design, synthesis and structural studies of meta-xylyl linked bis-benzimidazolium salts: potential anticancer agents against ‘human colon cancer’. Chemistry Central Journal. 2012;6(1):1-14.

5. Iqbal MA, Haque RA, Nasri SF, Majid AA, Ahamed MBK, Farsi E, et al. Potential of silver against human colon cancer:(synthesis, characterization and crystal structures of xylyl (Ortho, meta, & Para) linked bis-benzimidazolium salts and Ag (I)-NHC complexes: In vitro anticancer studies). Chemistry Central Journal. 2013;7(1):1-17.

6. Garcıa D, Escobar J, Bada N, Casquero J, Hernáez E, Katime I. Synthesis and characterization of poly (methacrylic acid) hydrogels for metoclopramide delivery. European polymer journal. 2004;40(8):1637-43.

7. Ahmadkhani L, Abbasian M, Akbarzadeh A. Synthesis of sharply thermo and PH responsive PMA-b-PNIPAM-b-PEG-b-PNIPAM-b-PMA by RAFT radical polymerization and its schizophrenic micellization in aqueous solutions. Designed monomers and polymers. 2017;20(1):406-18.

8. Motamedi S, Massoumi B, Jaymand M, Hamishehkar H. A dual stimuli-responsive star-shaped nanocarrier as de novo drug delivery system for chemotherapy of solid tumors. Journal of Polymer Research. 2020;27(9):1-12.

9. Gao Y, Li G, Zhou Z, Gao L, Tao Q. Sensitive complex micelles based on host-guest recognition from chitosan-graft-β-cyclodextrin for drug release. International journal of biological macromolecules. 2017;105:74-80.

10. Ghamkhari A, Rahdar A, Rahdar S, Susan MABH. Dual responsive superparamagnetic nanocomposites: Synthesis, characterization and adsorption of nitrate from aqueous solution. Nano-Structures & Nano-Objects. 2019;19:100371.

11. Adharis A, Ketelaar T, Komarudin AG, Loos K. Synthesis and self-assembly of double-hydrophilic and amphiphilic block glycopolymers. Biomacromolecules. 2019;20(3):1325-33.

12. Du J-Z, Tang L-Y, Song W-J, Shi Y, Wang J. Evaluation of polymeric micelles from brush polymer with poly (ε-caprolactone)-b-poly (ethylene glycol) side chains as drug carrier. Biomacromolecules. 2009;10(8):2169-74.

13. Wang D, Huan X, Zhu L, Liu J, Qiu F, Yan D, et al. Salt/pH dual-responsive supramolecular brush copolymer micelles with molecular recognition of nucleobases for drug delivery. RSC advances. 2012;2(31):11953-62.
